# Enzymatic and non-enzymatic pathways of kynurenines' dimerization: the molecular factors for oxidative stress development

**DOI:** 10.1371/journal.pcbi.1006672

**Published:** 2018-12-10

**Authors:** Aleksandr V. Zhuravlev, Oleg V. Vetrovoy, Elena V. Savvateeva-Popova

**Affiliations:** 1 Laboratory of Neurogenetics, Pavlov Institute of Physiology, Russian Academy of Sciences, Saint-Petersburg, Russia; 2 Laboratory of Regulation of the Brain Neuronal Functions, Pavlov Institute of Physiology, Russian Academy of Sciences, Saint-Petersburg, Russia; 3 Department of Biochemistry, Faculty of Biology, Saint-Petersburg State University, Saint-Petersburg, Russia; University of Houston, UNITED STATES

## Abstract

Kynurenines, the products of tryptophan oxidative degradation, are involved in multiple neuropathologies, such as Huntington's chorea, Parkinson's disease, senile dementia, etc. The major cause for hydroxykynurenines's neurotoxicity is the oxidative stress induced by the reactive oxygen species (ROS), the by-products of L-3-hydroxykynurenine (L-3HOK) and 3-hydroxyanthranilic acid (3HAA) oxidative self-dimerization. 2-aminophenol (2AP), a structural precursor of L-3HOK and 3HAA, undergoes the oxidative conjugation to form 2-aminophenoxazinone. There are several modes of 2AP dimerization, including both enzymatic and non-enzymatic stages. In this study, the free energies for 2AP, L-3HOK and 3HAA dimerization stages have been calculated at B3LYP/6-311G(d,p)//6-311+(O)+G(d) level, both in the gas phase and in heptane or water solution. For the intermediates, ionization potentials and electron affinities were calculated, as well as free energy and kinetics of molecular oxygen interaction with several non-enzymatically formed dimers. H-atom donating power of the intermediates increases upon the progress of the oxidation, making possible generation of hydroperoxyl radical or hydrogen peroxide from O_2_ at the last stages. Among the dimerization intermediates, 2-aminophenoxazinole derivatives have the lowest ionization potential and can reduce O_2_ to superoxide anion. The rate for O-H homolytic bond dissociation is significantly higher than that for C-H bond in non-enzymatic quinoneimine conjugate. However, the last reaction passes irreversibly, reducing O_2_ to hydroperoxyl radical. The inorganic ferrous iron and the heme group of Drosophila phenoxazinone synthase significantly reduce the energy cost of 2AP H-atom abstraction by O_2_. We have also shown experimentally that total antioxidant capacity decreases in Drosophila mutant *cardinal* with L-3HOK excess relative to the wild type *Canton-S*, and lipid peroxidation decreases in aged *cardinal*. Taken together, our data supports the conception of hydroxykynurenines' dual role in neurotoxicity: serving as antioxidants themselves, blocking lipid peroxidation by H-atom donation, they also can easily generate ROS upon dimerization, leading to the oxidative stress development.

## Author summary

Kynurenines, the products of tryptophan catabolism, are compounds with the multiple neuroactive properties. Hydroxykynurenines are redox modulators with a dual role in the oxidative stress development: being able to block lipid peroxidation, they also produce toxic free radicals during the oxidative self-dimerization. The later can occur both enzymatically and non-enzymatically. Computational study of the dimerization process may reveal the stages and intermediates being the source of the reactive oxygen species. This would help to provide therapeutic strategies aimed at the decrease of kynurenines' dimerization toxic effects. We have modeled oxidative conjugation of kynurenines using quantum-chemical approaches, performing calculations for the energies of the dimerization stages, as well as the redox properties of the intermediates. Phenoxazinone synthase provides a structural basis for the full oxygen reduction to water, prohibiting generation of toxic free radicals. The final non-enzymatic dimerization stages are the most probable source of hydroperoxyl and superoxide anion radical. The accumulation of L-3HOK in Drosophila mutant *cardinal* with impaired phenoxazinone synthase correlates with decrease in both total antioxidant capacity and lipid peroxidation in an age-dependent manner. Our results support the concept of protective role of proteins converting hydroxykynurenines to their dimeric forms, preventing free radicals overproduction and oxidative stress development.

## Introduction

The kynurenine pathway (KP) is a primary route of tryptophan degradation in mammals. Its metabolites, collectively called kynurenines, possess diverse neuroactive properties, being involved in the development of numerous neuropathologies, such as Alzheimer's disease (AD), Parkinson's disease (PD), Huntington's disease (HD), etc. [1–4]. Indoleamine-2,3-dioxygenase (IDO) or tryptophan-2,3-dioxygenase (TDO) governs the initial rate-limiting stage of KP, converting tryptophan to N-formylkynurenine, which is then non-enzymatically hydrolyzed to kynurenine. Till the late 1970^th^, NAD^+^ synthesis was thought to be the only biological role for KP, however, now its neuro- and immunomodulatory role is well-established [5]. Kynurenines are deeply involved in neurotoxicity, neuroinflammation and excitotoxicity. These three phenomena result from interrelationship of metabolic disturbances leading to increase in reactive oxygen and nitrogen species (ROS, RNS) [6].

There are two chief molecular mechanisms by which kynurenines act on nervous system: the ligand-receptor interaction and the regulation of oxidative stress. Kynurenine (KYN), the first of KP metabolites, is a ligand of the aryl hydrocarbon receptor, suppressing antitumor immune response, both in humans [7] and Drosophila [8]. Quinolinic acid (QUIN) is an agonist and kynurenic acid (KYNA) is an antagonist of ionotropic glutamate receptors [9,10], modulating neurodegenerative processes development [11]. KYNA is also a ligand for G protein-coupled receptor GPR35, regulating Ca^2+^ mobilization and inositol phosphate production [12]. L-3-hydroxykynurenine (L-3HOK) and 3-hydroxyanthranilic acid (3HAA) are antioxidants inhibiting peroxyl radical-mediated oxidation of phosphatidylcholine and plasma lipid peroxidation [13,14]. At the same time, their autoxidation leads to the hyperproduction of noxious ROS, damaging cellular structures and causing apoptotic cell death [15,16].

Both prooxidant and antioxidant properties of hydroxykynurenines are due to the easiness of one-electron or H-atom abstraction. 2-aminophenol (2AP), a structural precursor of L-3HOK and 3HAA, has a significantly lower homolytic O-H bond dissociation enthalpy (BDE) compared to that for phenol [17]. Antioxidants should have O-H BDE low enough to provide free radicals quenching by H-atom. However, too low BDE may cause the antioxidant H abstraction by molecular oxygen, converting both of them to free radicals able to initiate lipid peroxidation [18]. There must be an optimal balance between the ability to donate H-atom and chemical inertness of the compound to remain harmless to a cell. The antioxidant protection systems, including specific enzymes, govern ROS and other free radicals conversion to the non-active low-energy compounds, such as water molecule.

**Fig 1:**
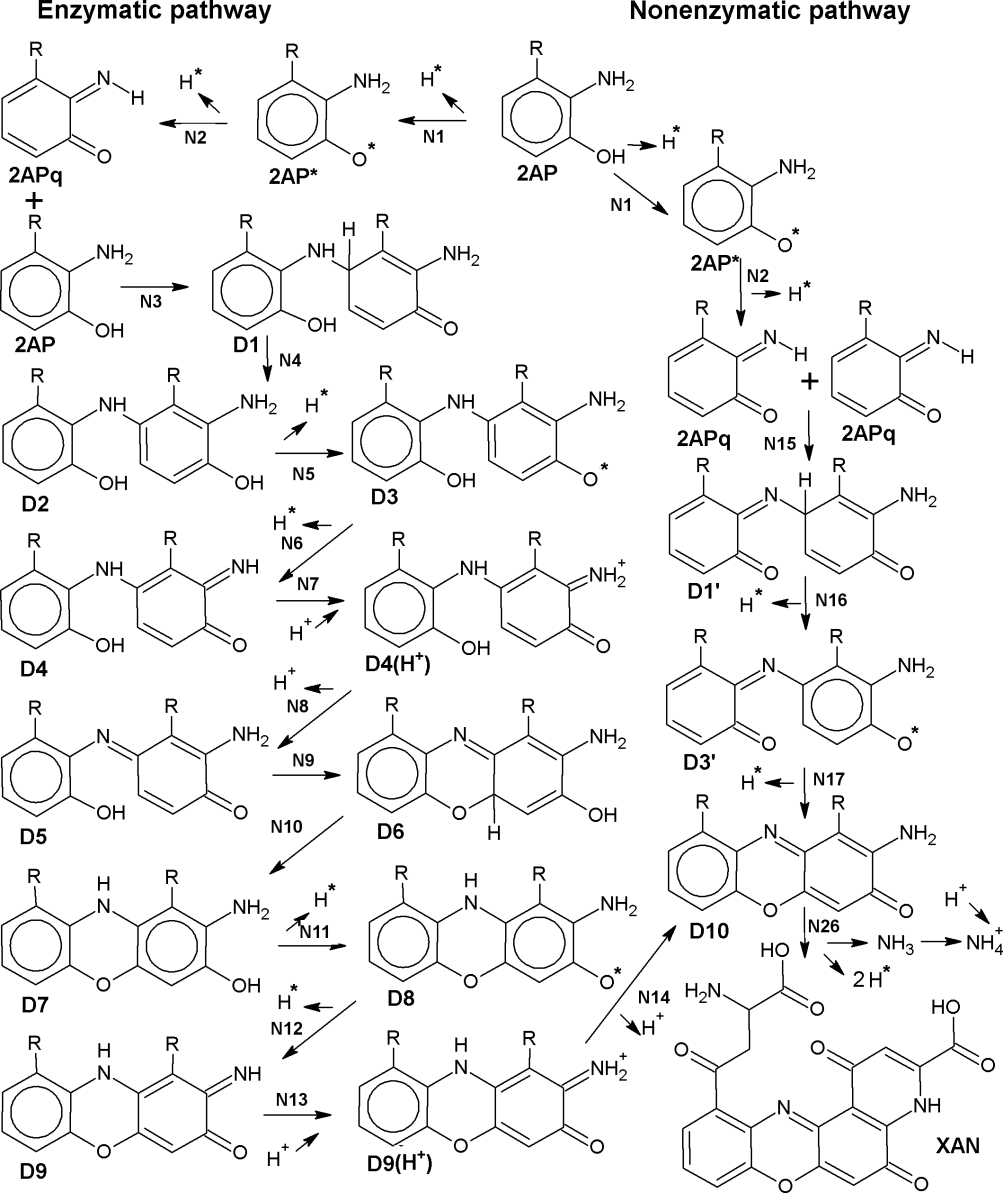
R-2-aminophenol (R-2AP) enzymatic and non-enzymatic conversion to phenoxazinone structure. Here and after: D1-10 are R-2AP dimers formed according to scheme given in [19] with some modifications; D1'and D3' are R-2AP dimers non-enzymatically formed according to scheme given in [20]. N–the number of reaction. See also Materials and Methods.

**Fig 2:**
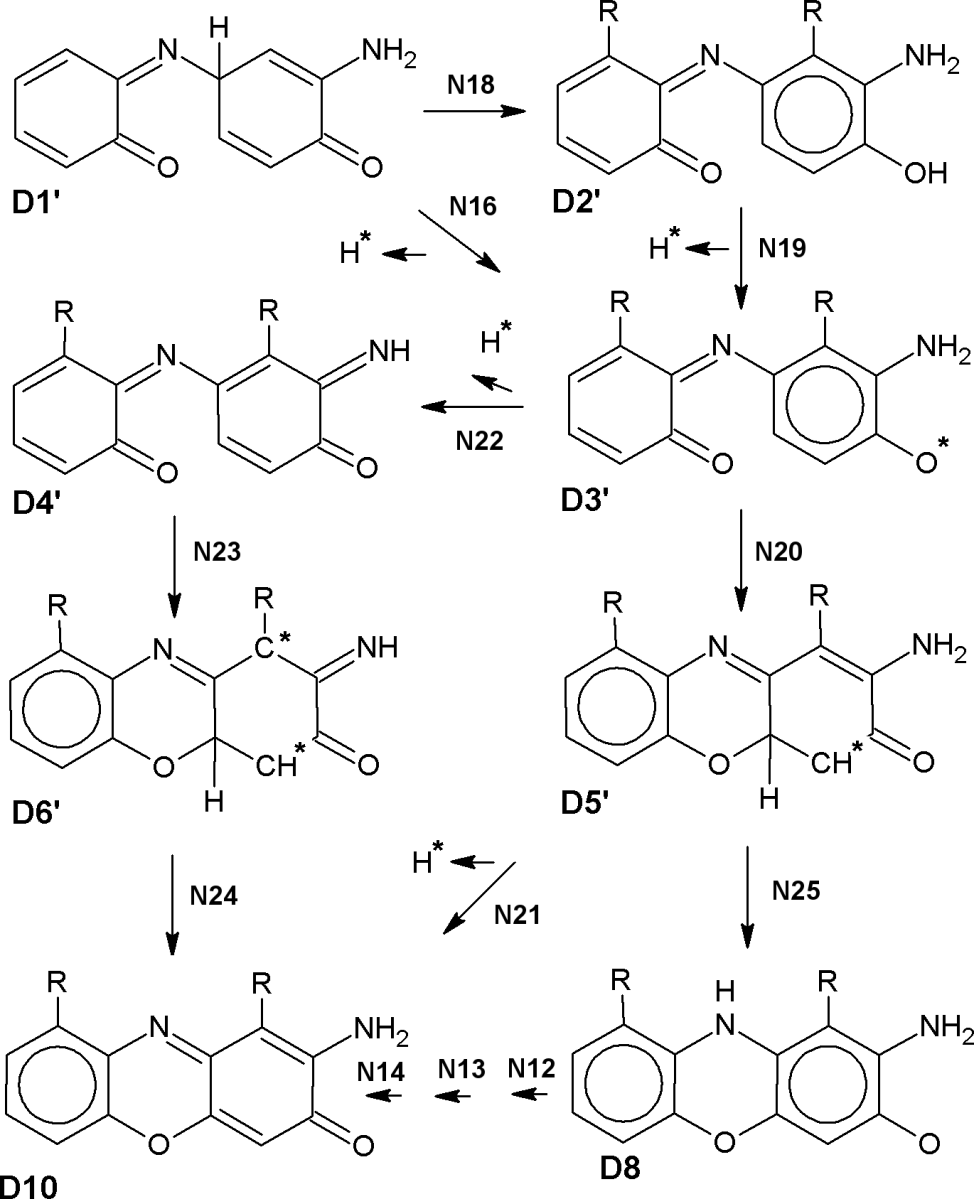
Alternative stages of R-2AP non-enzymatic dimerization. D2' and D4'–D6' are hypothetical non-enzymatically formed intermediates.

2AP dimerization can occur enzymatically [19] and non-enzymatically [20,21] being catalyzed by several organic or inorganic compounds. In both cases, the condensation of two 2AP (or their structural analogues R-2AP) includes three consecutive stages of two H-atom abstraction equal to six-electron oxidation (Fig 1). Likewise, 3HAA oxidative dimerization by laccase leads to cinnabaric acid (CIN) formation [22]. Non-enzymatic dimerization may occur via several redundant stages. Here, we present the scheme of 2AP non-enzymatic conversion including both experimentally found [20] and some hypothetic intermediates (Fig 2). Being different in their intermediates and the number of stages, both enzymatic and non-enzymatic pathways lead to formation of 2-aminophenoxazinone (2APX) or its structural analogue 2R-2APX.

The summary equation is the following:

$$R-2AP+O_{2}+oxidant\to R-2APX-R+2H_{2}O+H_{2}-oxidant$$ (1)

where the last two H-atom abstractions are considered to be a non-enzymatic process [19,21,23]. For L-3HOK, there is an additional stage of two H-atom abstraction from R-2APX-R leading to the fourth planar ring cyclization and formation of xanthommatine (XAN), the insect brown pigment [24].

Phenoxazinone synthases (PHSs, class EC 1.10.3.4) belong to various structural types of proteins having different functions in bacteria, plants, fungi, and animals. phsA, the first reported PHS involved in actinomycin production by *Streptomyces antibioticus*, is an oligomer Cu^2+^-containing enzyme that shares structural similarities to laccase [19,21]. It uses 2AP and O_2_ as substrates, hydrogen peroxide may be an intermediate of the reaction. In *Drosophila melanogaster*, PHS is a phsA functional analogue encoded by *cardinal (cd)*, showing significant similarity to the heme-containing peroxidase [25]. It catalyzes the conversion of 3HOK to XAN and produces CIN from 3HAA, its most specific substrate [26]. Some other proteins, such as laccase, tyrosinase, peroxidase and human hemoglobin are able to catalyze similar reactions [21].

In Drosophila *cd* mutant, PHS activity is decreased to ~40% of the wild type [24]. Enzymatic dihydroxanthommatin (DXAN) synthesis from 3HOK is disrupted in *cd* demonstrating 2.9-fold 3HOK increase in heads [24,27], as well as age-dependent memory loss [28]. The oxidative dimerization of 3HOK and 3HAA to XAN and CIN can occur both enzymatically and non-enzymatically. Spontaneous XAN and CIN formation in Drosophila homogenates is catalyzed by themselves serving as electron acceptors, while DXAN is a powerful inhibitor of the oxidation process. Unexpectedly, the enzymatic activity, possibly associated with some unspecific oxidase, was shown to be similar in *cd* and two other kynurenine pathway mutants, *vermilion* and *cinnabar*, and non-enzymatic oxidation activity was depleted in *cd* [29]. The last one was probably associated with XAN which autocatalyzes its own formation.

Both activities seem to produce an impact on age-dependent progressive *cd*-specific loss of middle-term memory accompanied by neurodegeneration [28] and male courtship song distortions [30,31]. Drosophila *cd* mutant is a model object to study senility processes and ROS-induced neurodegeneration mechanisms at the molecular level [28]. To develop the therapeutic strategy implying natural and synthetic antioxidants, we should properly understand the impact of enzymatic and non-enzymatic R-2AP conversion pathways to oxidative stress progression. The by-products of enzymatic pathway are two chemically inert water molecules and some unknown reduced oxidant. At the same time, non-enzymatic oxidation likely leads to toxic ROS formation due to single electron or H-atom abstraction by molecular oxygen. PHS dysfunctions should shift the balance to the non-enzymatic oxidation, which in turn may occur via several pathways.

Studying the redox properties of dimerization intermediates would reveal the most prone to produce ROS. The complex nature of dimerization processes makes it hard to study all its separate stages experimentally. This requires the usage of computational and modelling approach. Previously, the ability of hydroxykynurenines to donate H-atom and inhibit phenoxyl or methyl peroxy radicals was computationally investigated [32]. Both L-3HOK and 3HAA are powerful antioxidants, rapidly quenching peroxy radicals by hydroxyl H-atom transfer. In this study, we use similar approach to estimate Gibbs free energies (ΔG) of R-2AP dimerization stages for enzymatic and non-enzymatic pathways. The redox properties of R-2AP dimerization products were estimated by calculating their ionization potentials (IP) and electron affinities (EA). ΔG and rates of H-atom abstraction by molecular oxygen were calculated for some intermediates of non-enzymatic pathway. *D*. *melanogaster* PHS structure in complex with R-2AP was modeled and the energies of 2AP interaction with PHS oxy-heme group were calculated. For all compounds, single-point energies were calculated both in the gas phase and in heptane or water solution modeling hydrophobic lipid and aqueous surrounding, respectively. Finally, the age-dependent total antioxidant capacity (TAC) and lipid peroxidation level (LP) were estimated in Drosophila *cd[1]* strain with L-3HOK accumulation.

## Results

### Free energies of R-2AP dimerization via enzymatic and non-enzymatic pathways

The enzymatic and non-enzymatic R-2AP dimerization pathways share the common stages of initial H-atom dissociation from R-2AP (N1, 2) leading to quinoneimine (R-2APq) production. Enzymatic pathway includes the following stages: conjugation (N3, 9); H-atom abstraction (5, 6, 11, 12); isomerization/ H-atom migration (N4, 10), proton association (N7, 13); proton dissociation (N8, 14) (Table 1). We called the “classic” pathway of dimerization the way including stages N1 –N14, firstly described for 2AP in [19]. Non-enzymatic pathway may exert via several redundant stages, such as conjugation (N15, 20, 23), H-atom abstraction (N16, 17, 19, 21, 22), isomerization/ H-atom migration (N18, 24, 25) (Table 2). For L-3HOK-D10, there is an additional non-enzymatic conjugation stage (N26) leading to XAN formation and two H-atom abstractions. Noteworthy, not all the stages of the “enzymatic pathway” are catalyzed by PHS. For instance, D7 → D10 reaction may occur spontaneously [19]. Hence, the very distinction of “enzymatic” and “non-enzymatic” dimerization pathways is rather conditional.

**Table 1:** Free energies (ΔG, kcal/mol) of R-2AP dimerization via the enzymatic pathway.

		Gas				Heptane				Water			
N	Half-reaction	2AP	L-3HOK	3HAA	3HAAi	2AP	L-3HOK	3HAA	3HAAi	2AP	L-3HOK	3HAA	3HAAi
1	R-2AP → R-2AP*+ H*	58.230	59.028	58.689	48.361	55.894	57.757	57.310	49.204	55.914	58.817	58.320	54.274
2	R-2AP* →R-2APq+ H*	64.400	67.063	72.711	70.811	67.302	69.382	74.670	72.947	69.402	70.022	74.510	72.607
3	2AP + 2APq→ D1	5.799	5.798	3.095	37.676	6.818	8.351	6.019	18.321	7.108	7.291	7.779	-0.199
4	D1 → D2	-12.182	-10.994	-13.769	6.477	-12.704	-10.919	-15.538	3.431	-14.334	-11.039	-16.938	-4.039
5	D2 → D3 + H*	53.284	53.127	52.114	39.317	50.953	51.075	50.664	40.584	50.623	51.315	51.294	46.904
6	D3 → D4 + H*	64.235	61.272	70.485	64.680	67.070	63.903	72.002	68.423	69.250	65.843	71.492	72.493
7	D4 + H^+^→ **D4 (H**^**+**^**)**^**$**^	-239.819	-230.274	-241.921	-372.143	[-8.341]	[-7.128]	[-10.699]	[-3.520]	-10.428	-1.299	-9.277	-17.979
8	D4 (H^+^) → D5+ H^+^	230.936 [-8.876]	222.443[-7.831]	229.487 [-12.434]	370.459 [-1.684]					3.197 [-7.231]	-5.319 [-6.618]	1.788 [-7.489]	11.619 [-6.360]
9	D5 → D6	6.211	5.273	12.539	36.516	5.775	3.509	10.986	30.554	5.485	3.199	7.856	16.334
10	D6 → D7	-16.125	-19.180	-28.209	-38.745	-16.032	-17.834	-27.015	-36.704	-16.072	-15.064	-23.525	-29.964
11	D7 → D8 + H*	50.631	51.287	49.565	35.053	48.166	49.646	48.050	36.181	47.326	49.746	48.600	42.591
12	D8 → D9 + H*	60.238	54.177	66.544	65.835	62.874	56.955	68.334	68.114	64.904	58.295	67.754	68.024
13	D9 + H^+^→ **D9 (H**^**+**^**)**	-235.127	-227.299	-236.913	-376.557	[-14.643]	[-1.326]	[-5.480]	[4.886]	-8.572	4.544	-7.148	-14.534
14	D9 (H^+^) → D10 + H^+^	219.543 [-15.584]	225.868 [-1.431]	231.321 [-5.592]	382.735 [6.178]					-4.131 [-12.703]	-5.720 [-1.176]	1.778 [-5.370]	15.350 [0.816]
	SUM	310.254	317.589	325.738	370.475	313.132	323.371	329.303	352.421	319.672	330.631	334.283	333.481

SUM: the sum of ΔG for half-reactions 1–14. Bold: the optimization and Hessian calculation of protonated (H^+^) forms were performed in water solution.

$: structure is fluctuating around the local minimum, the optimization failed to converge. In square brackets: the summary ΔG for N7 and N8.

**Table 2:** Free energies (kcal/mol) of R-2AP dimerization via the non-enzymatic pathway.

		Gas				Heptane				Water			
N	Half-reaction	2AP	L-3HOK	3HAA	3HAAi	2AP	L-3HOK	3HAA	3HAAi	2-AP	L-3HOK	3HAA	3HAAi
15	R-2APq+ R-2APq → D1 '	5.428	2.868	-5.040	58.242	6.752	5.432	-2.399	35.867	6.932	5.702	-0.209	9.317
16	D1 ' → D3 ' + H*	35.327	39.935	39.980	33.985	33.408	38.635	38.624	31.403	33.628	38.125	38.734	31.613
17	D3 ' → D10 + H*	24.239	22.606	27.997	39.903	26.580	25.026	29.118	40.849	28.480	29.126	30.098	38.789
18	D1 ' → D2 '	-15.386	0.726	-8.522	-0.379	-15.931	-0.344	-9.522	-3.639	-16.271	-4.994	-11.032	-10.129
19	D2 ' → D3 ' + H*	50.713	37.503	48.502	34.364	49.339	35.929	48.146	35.042	49.899	43.119	49.766	41.742
20	D3 ' → D5'	5.190	4.041	8.872	14.155	5.105	1.883	7.735	13.768	5.405	3.533	7.455	12.228
21	D5 ' → D10 + H*	19.049	20.270	19.125	25.749	21.475	23.143	21.383	27.081	23.075	25.593	22.643	26.561
22	D3 ' → D4' + H*	71.086	68.879	77.053	75.047	73.744	69.126	79.402	87.462	74.794	70.766	79.412	86.462
23	D4 ' → D6'	11.885	21.032	12.269	18.802	7.935	17.277	7.251	8.108	8.645	17.377	6.901	4.088
24	D6' → D10	-58.732	-65.600	-61.325	-53.945	-55.099	-61.377	-57.535	-54.721	-54.959	-59.017	-56.215	-51.761
25	D5 ' → D8	-25.605	-32.475	-41.827	-46.264	-26.756	-32.486	-41.471	-45.919	-29.126	-31.526	-39.741	-42.279
26	D10 → XAN + NH4^+^ + 2H*		144.553			108.357				76.563			
	D10 → XAN + NH3 + 2H*		85.749			85.895				84.415			

**Fig 3:**
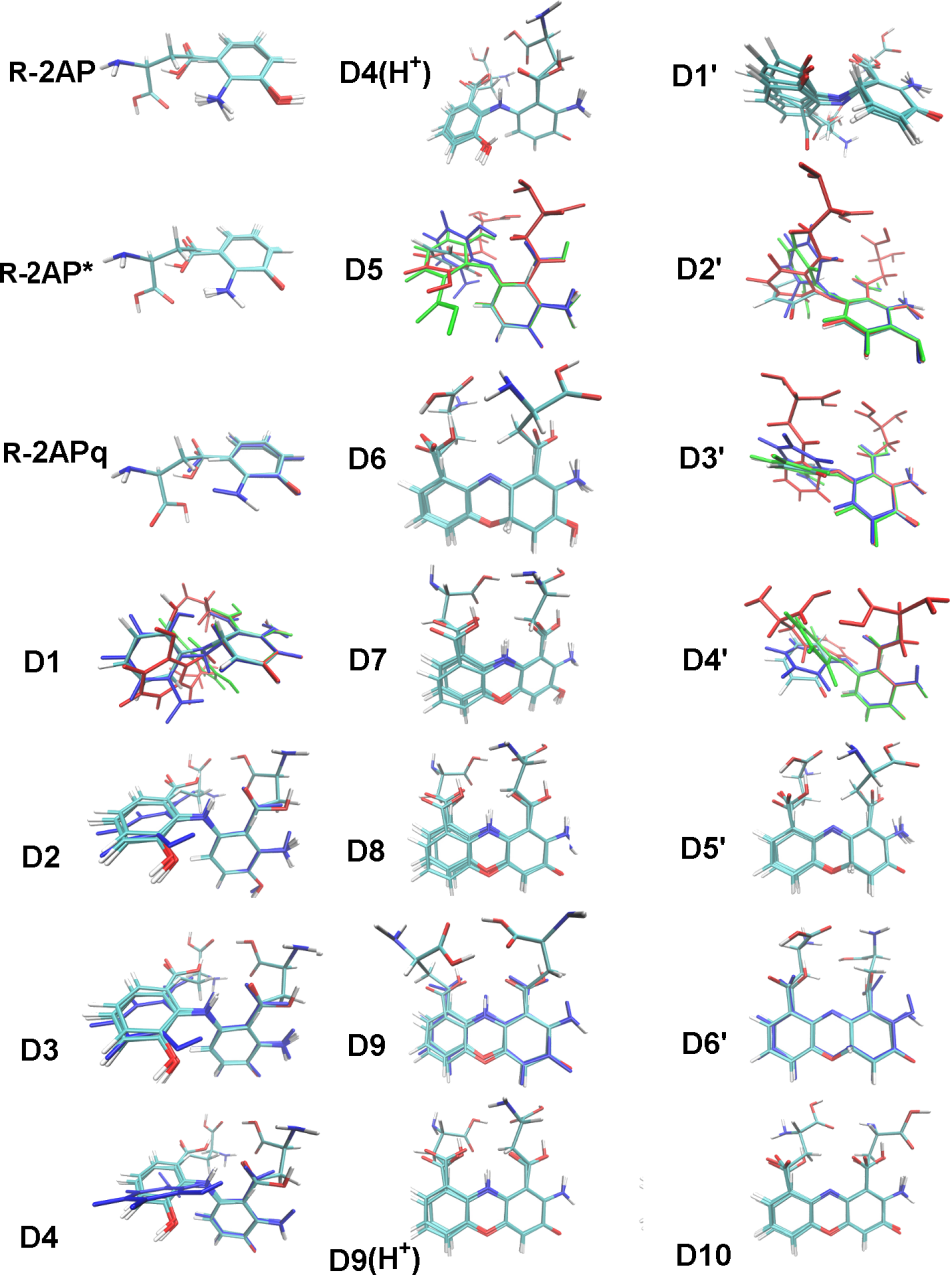
Alignment of R-2AP dimerization products. Color scheme: for atoms: C–cyan, O–red, N–blue, H–white; for structures with significant conformation deviations: red–L-3HOK, green– 3HAA, blue– 3HAAi.

For the majority of the stages, the optimal conformations of dimer skeleton including two connected planar rings with OH and NH_2_ groups are similar among all the studied compounds, though the side chain conformations may vary (Fig 3). Hence, we consider our models of R-2AP dimerization intermediates to be generally correct. However, for some structures the difference between R-2AP-D is pronounced, e.g. for D1, D5 and D2'-D4'. Mainly this is due to hydrogen bonds (HBs) formation by side chains of several compounds. 2AP lacking R side chains has a lower opportunity to form intramolecular HBs or electrostatic interactions, positively or negatively shifting ΔG values. Noteworthy, at physiological pH L-3HOK is in zwitterion (zi) form. This may significantly affect its side chains interactions, e.g. leading to NH_3_^+^ and COO^-^ groups’ spatial approach. We found L-3HOKzi and its dimers to be unstable in the gas phase and poorly converging to the local minimum *in aqua* that made impossible to use them in our computations.

The total ΔG for two 2APs conversion to 2APX (D10) is the same for the both pathways (shown in Table 1). In the gas phase, its values are close for the uncharged compounds (2AP, L-3HOK, 3HAA; 325.739±12.137 kcal/mol), being significantly lower than that for the negatively charged 3HAAi. The aforementioned difference becomes lower in heptane and actually disappears in water solution. Here, ΔG is almost identical for three kynurenines (332.798±4.960 kcal/mol), being higher compared to that for 2AP. The conjugation stages (N3, 15) are slightly endothermic or exothermic (for 3HAA, N15). Therefore, they may occur spontaneously. For 3HAAi, N3 conjugation is highly endothermic in the gas phase or in heptane. Seemingly, this is due to the repulsion of two negatively charged COO^-^ groups, which becomes more energetically favorable in water solution. The intramolecular conjugation stages N9 and N20 are also slightly endothermic, with the highest ΔG for 3HAAi-D, while N24 stage is less energetically favorable than N20. Taking into account BSSE correction, the dimerization energies should be ~3–7 kcal/mol higher, and 3HAAi dimerization is even more energetically unfavorable compared to the uncharged form (see Materials and Methods). The tautomerization stages (N4, 10, 18) following the conjugation stages and leading to the second ring aromatization are mainly exothermic. For 2AP, L-3HOK and 3HAA, ΔG values are strongly correlated both in the gas phase and in heptane or water solution, while for 3HAAi the correlation is strong only *in aqua* (Table I in S2 Table).

H-atom homolytic bond dissociation stages have the highest ΔG values (BDG): the average BDG is 50.056±5.136 kcal/mol for the gas phase, 51.032±5.365 kcal/mol for heptane solution, and 52.335±5.229 kcal/mol for water solution (the differences are non-significant; n = 44). H-atom abstraction BDG widely vary for the different stages, being maximal for N22 (~76 kcal/mol) and minimal for N21 (~23 kcal/mol) (S1 Fig). The only exception is the hypothetical reaction D6' → D10 (N24) where BDG is highly negative. D6' is a biradical and triplet in its ground state, so its spontaneous transition to the closed shell D10 is spin-forbidden. Hence, the whole D3' → D4' → D6 → D10 pathway (N22, 23, 24) seems to be complicated. The alternative pathway includes D5' intermediate which is either directly converted to D10 (N21), or isomerized to D8 (N25), joining the “classic” pathway (see Fig 2).

For N-H bond dissociation leading to quinoneimine structures formation, BDG is significantly higher than that for O-H bond dissociation (compare N22 –N19, N2 –N1, N6 –N5, N12 –N11). Partially, this may be caused by the addition of a diffuse orbital to O-atom at level III (see Materials and Methods). However, the average difference between N-H and O-H BDG calculated at level II is 11.081±4.854 kcal/mol, while for the bond dissociation enthalpy (BDE) it is 12.798±6.773 kcal/mol (n = 12, enzymatic pathway). Hence, N-H bond is more difficult to break, especially for 3HAAi-D. BDG is lower for dimers compared to monomers (compare N5 –N1, N6 –N2, N19 –N1) and for the oxidized dimers compared to the reduced dimers (compare N11 –N5, N12 –N6). This fact corresponds to proposal of [19]: 2-aminophenols become more electron rich as the oxidation progresses, allowing for non-enzymatic oxidation at the last stage. At physiological pH, D4 and D9 are mainly in the protonated (NH_2_^+^) form. We modelled D3 → D5 and D8 → D10 conversions as consisting of three stages: a. aromatic N-H bond dissociation (N6, 10); b. quinoneimine NH group protonation (N7, 11); c. charge migration and amine bridge NH group deprotonation (N8, 12). For b. and c., the summary ΔG is negative, except for 3HAAi-D9(H^+^).

For non-enzymatic pathway stages where C-H bond dissociation is accompanied by the second ring aromatization (N16) or intramolecular cyclization (N17), BDG is lower than that for O-H BDG values, except for 3HAAi-D in N17. However, C-H bond dissociation rate may be low due to the high value of the activation barrier. In this case, N16 can proceed in two stages: the exothermic keto-enol tautomerization of D1' second ring (N18) and subsequent enol O-H bond dissociation (N19) with BDG close to those for the similar dissociation step of enzymatic pathway (N5). As mentioned before, N17 is likely to proceed via intramolecular cyclization (N20) and subsequent C-H bond dissociation (N21). N21 has the lowest BDG, making D5' a powerful H-atom donor in reactions with molecular oxygen or some other ROS generators. However, D5' C-H bond dissociation may be kinetically inhibited, as well as that for D1'.

O-H BDG slightly differs for the kynurenines' derivatives: typically, it is somewhat higher for L-3HOK-D compared to that for 3HAA-D (N1, N5, N11), except in N19. In this case, low BDG value may be explained by the relaxation of the distorted L-3HOK aromatic ring (see below). Contrary, N-H BDG is higher for 3HAA compared to all the other compounds. For 3HAAi-D, O-H BDG is significantly lower compared to the non-ionized form in the gas phase, the difference becoming less pronounced in water solution. This corresponds to our previous data that negatively charged carboxylic group decreases O-H bond dissociation energy in kynurenines–an effect reduced in media with the high dielectric constant [32]. This is similar for N-H bond: BDG becomes the same or even higher for 3HAAi-D compared to 3HAA-D in water solution. On the contrary, C-H BDG is higher for 3HAAi-D than for 3HAA-D (N17, N21). However, in all cases BDG differences for kynurenines' derivatives are statistically insignificant (S1 Table).

The other probable factors causing ΔG differences for 2AP and kynurenines' dimerization are:

1. HB formation: 3HAAi-D (N6 stage, COO^-^‥ OH: HB), 3HAA/3HAAi-D (N10, NH‥ COOH/COO^-^: 2 HBs), L-3HOK-D (N10, NH…CO: HB), L-3HOK-D (N12, NH‥ CO: HB)–ΔG decreases, as a result.
2. HB break: 3HAAi-D (N4, COO^-^‥ OH: HB), 3HAA/3HAAi-D (N14, NH…COOH/COO: 2 HBs), L-3HOK-D (N13, NH…CO: HB), L-3HOK-D (N14, NH…CO: HB)–ΔG rises;
3. Repulsion of charged groups: 3HAAi-D (N3, 9, 15, 20: COO^-^ groups)–ΔG rises.
4. Protonation of negatively charged compounds: 3HAAi-D (N7, 13)–ΔG decreases.
5. Deprotonation of neutral compounds: 3HAAi-D (N8, 14)–ΔG rises.
6. Aromatic ring distortion: L-3HOK-D (N18, the first ring); 3HAA/3HAAi-D (N22, the second ring)–ΔG rises.
7. Aromatic ring relaxation: L-3HOK-D (N19, the first ring)–ΔG decreases.

The effects of polar interactions are decreased in heptane and are nearly extinguished in water solution.

In living organism, 2AP derivatives form complexes with metal ions such as Cu^2+^ affecting their redox properties [33]. In this study, we are focused on H-atom donation ability of R-2AP derivatives in apo form, without inorganic ions, seeking stages with the lowest H-atom abstraction BDG, which may partly redox molecular oxygen (O_2_) to toxic ROS. In addition, we studied the electron-donating and electron-accepting capacities of R-2AP derivatives in free form, without metal ions and any other substances modulating their redox properties.

### Free energies of oxygen species formation during R-2AP oxidation

The total energy effect of R-2AP dimerization is determined by Eq 1: six H-atoms are abstracted by O_2_ and some oxidant to produce two H_2_O molecules and some reduced oxidant. In Table 3, ΔG is given for the summary reaction while considering O_2_ to be an oxidant abstracting the last two H-atoms. In this case, the summary dimerization reaction is thermodynamically favorable, with a total energy output of ~50–100 kcal/mol. However, O_2_ may be only partly reduced at some stages, forming ROS, such as hydrogen peroxide (H_2_O_2_), hydroperoxyl radical (HO_2_*) or superoxide anion radical (O_2_*^-^). O_2_*^-^ formation is a result of an electron abstraction by O_2_ (see below). HO_2_* and H_2_O_2_ can be formed through a single- and paired H-atom abstraction, respectively. Likely, this occurs in compounds where the aromatic OH and NH_2_ groups are oriented towards O_2_ by their H-atoms. The appropriate pair half-reactions would be N1+2, 5+6, 11+12, and 19+22. The half-reactions of R-2AP-D oxidation corresponding to the summary reactions with ΔG<0 are shown in Table 3.

**Table 3:** Free energies (kcal/mol) for half-reactions of O_2_ with H-atom of R-2AP-D and for the reduction to O2*^-^.

O_2_ reduction		R-2AP oxidation
Half-reactions	ΔG	Half-reaction with ΔG<0 for the overall reaction
Gas		
1.5 O_2_+6H* → 3H_2_O	-416.754	SUM (all)
O_2_ + 2H*→ H_2_O_2_	-108.188	5+6(4);11+12(2, 4); 19+22(2)
O_2_ + H* → HO_2_*	-37.988	11(4); 16(1, 4); 17(1–3); 19(2, 4); 21
O_2_ → O_2_*^-^	-13.945	for 3HAAi: D2, 3, 4, 7, 8, 3'
Heptane		
1.5 O_2_+6H* → 3H_2_O	-415.782	SUM (all)
O_2_ + 2H*→ H_2_O_2_	-105.873	11+12(4); 19+22(2)
O_2_ + H* → HO_2_*	-37.687	11(4); 16(1, 4); 17(1–3); 19(2, 4); 21
O_2_ → O_2_*^-^	-85.83	for 3HAAi: D1, 2, 3, 4, 5, 6, 7, 8, 9, 10, 1', 3'
Water		
3O_2_+6H* → 3H_2_O	-431,358	SUM (all)
O_2_ + 2H*→ H_2_O_2_	-111.033	11+12(2, 4), 26
O_2_ + H* → HO_2_*	-41.477	16; 17; 21
O_2_ → O_2_*^-^	-87.31	

Before brackets–the number of a half-reaction in Tables 1 and 2 (N). In brackets–the number of compound: 1. - 2AP-D, 2.—L-3HOK-D, 3. - 3HAA-D, 4. - 3HAAi-D. SUM–the summary reaction of R-2AP dimerization to D10 with O_2_ as electron acceptor; all–all compounds.

For L-3HOK and 3HAAi, H_2_O_2_ can be formed at the stages N11+12, believed to pass spontaneously or outside the catalytic center of PHS [19]. In the non-aqueous phase, H_2_O_2_ formation may occur via non-enzymatic oxidation of D2' to D4' (N19+22). In the gas phase, it is also possible for the reactions N5-6, which normally pass within the catalytic center. For 2AP-D and non-ionized 3HAA-D, the same reactions are thermodynamically unfavorable. For L-3HOK-D10 in water solution, the final two H-atom abstraction leading to XAN (N26) can be accompanied by H_2_O_2_ production. HO_2_* formation is the least sterically hindered one, as it occurs via a single H-atom abstraction. However, for the “classic” pathway, it is energetically favorable only for N11 with 3HAAi (in gas and heptane). For the non-enzymatic pathway, both C-H (N16, 17, 21) and O-H (N19, in gas and heptane) dissociation energies are low enough to provide H-atom migration to O_2_. To sum up, non-enzymatically formed R-2AP-D are more thermodynamically prone to interact with O_2_ leading to ROS formation.

### Ionization potentials, electron affinities, and electronegativities of R-2AP derivatives

The redox properties of R-2AP-D are not restricted to H-atom abstraction power, but also related to their ability to donate and receive electrons. Here, we limited our calculations to forms that were shown to exist experimentally (see Fig 1), except for the protonated forms which IP are significantly higher due to their positive charge.

IP widely varies for different R-2APs, depending on both their chemical nature and dimerization form (Table 4 and S2 Fig). In the gas phase, 3HAAi-D have significantly lower IP compared to the other compounds, such as 3HAA-D (S1 Table), in accordance to previous data that the ionized acidic group decreases kynurenines' IP [32]. This effect is partly compensated in heptane solution and only to a small extent remains in water solution. IP is slightly lower for 2AP-D compared to L-3HOK-D, as well as to 3HAA-D. Thus, in the aqueous surroundings, the influence of R-2AP-D chemical nature on its electron-donating power becomes stronger and the influence of its acid-base forms on IP values weakens.

**Table 4:** The ionization potentials (IP) of R-2AP-D (kcal/mol).

N		Gas				Heptane				Water			
		2AP	L-3HOK	3HAA	3HAAi	2AP	L-3HOK	3HAA	3HAAi	2AP	L-3HOK	3HAA	3HAAi
	Common intermediates												
1	2AP	164.117	162.258	169.726	**74.475**	138.501	140.417	144.833	**107.384**	115.721	120.737	121.873	**130.254**
2	2AP*	169.569	164.544	173.667	**109.935**	147.295	145.826	151.607	**101.612**	122.955	124.966	126.967	**119.232**
3	2APq	**225.274**	187.688	206.433	106.970	**182.763**	163.596	181.238	127.181	**155.263**	141.026	157.728	150.671
	Enzymatic pathway												
4	D1	156.054	162.435	161.459	**30.600**	134.580	142.910	140.259	**43.143**	115.420	123.090	120.459	**118.883**
5	D2	142.385	150.010	146.307	1.699	122.551	129.424	126.682	52.704	106.381	111.004	109.632	111.186
6	D3	139.983	146.549	144.102	**0.957**	123.507	130.270	127.607	**51.781**	107.087	112.580	110.387	**103.471**
7	D4	166.378	169.757	172.613	20.420	143.980	151.663	152.408	69.743	123.160	136.193	133.798	124.433
8	D5	159.845	165.277	165.407	14.903	139.689	145.243	145.302	63.685	122.109	125.553	127.202	117.805
9	D6	151.876	160.487	161.285	**12.474**	131.272	143.122	140.982	**59.322**	113.532	127.202	123.182	107.582
10	D7	137.538	141.940	142.197	-3.135	117.519	123.958	122.506	43.822	100.099	107.858	104.706	94.892
11	D8	140.693	142.364	145.311	**2.965**	123.214	112.536	127.942	**51.915**	104.534	110.976	108.902	**99.715**
12	D9	173.081	174.437	180.871	**24.029**	150.194	155.376	159.439	**71.278**	127.244	139.246	138.649	**121.068**
13	D10	170.510	168.494	177.467	15.375	149.212	148.562	156.456	64.426	128.922	130.882	136.526	117.186
	Non-enzymatic pathway												
14	D1'	170.472	171.145	178.118	19.180	148.452	152.327	157.187	68.410	128.142	134.087	138.877	126.490
15	D3'	145.886	152.647	154.275	12.897	129.193	138.120	137.437	64.334	110.863	122.410	119.677	116.354
16	XAN		178.983				158.372				140.052		

Bold: the cationic forms were optimized in water solution.

For the most compounds, R-2APq and 2-aminophenoxazinole derivative D7 have the highest and the lowest IP, respectively. Quninoneimines possess lower ability to abstract an electron compared to the radical forms of R-2AP and their dimers (compare R-2APq–R-2AP*, D9 –D8, D4 –D3). Forms with H-atom attached to the second ring disrupting its aromatic system have higher IPs compared to forms with two aromatic rings (compare D1 –D2, D6 –D7), possibly, due to the lower capacity for the unpaired electron delocalization. The oxidation of O-H and N-H groups disrupting the ring aromaticity decreases the ability to abstract an electron (compare D10 –D7, D9 –D7, D4 –D2, D1'–D1, D3'–D3). At the same time, IP decreases when rings conjugate keeping their aromaticity (R-2AP–D2 –D7).

For the same compounds, EA were calculated (Table 5 and S3 Fig). Predictably, the negatively charged 3HAAi derivatives have the most negative EA, which is lower for the dimers with two ionized carboxylic groups than for the monomer. This difference decreases in heptane and virtually vanishes in water solution. EA is slightly less for 2AP-D compared to L-3HOK-D, as well as to 3HAA-D. Thus, in the aqueous surroundings, EA variations for different compounds become less pronounced. Among the uncharged compounds, EA is maximal for D3'. The trend for electron accepting power change is mirror to that for the electron donating ability, as EA decreases within the following groups: D3'–D3, D1'–D1, D4 –D2, D10 –D7, D9 –D7; D1 –D2, D6 –D7; R-2APq–R-2AP*–R-2AP, D9 –D8, D4 –D3. However, the uncharged R-2AP has lower EA compared to D2, and D7 is intermediate between them. All these three substances preserve 2AP moiety, being the worst electron acceptors among the studied compounds.

**Table 5:** The electron affinity (EA) of R-2AP-D (kcal/mol).

N		Gas				Heptane				Water			
		2AP	L-3HOK	3HAA	3HAAi	2AP	L-3HOK	3HAA	3HAA i	2AP	L-3HOK	3HAA	3HAA i
	Common intermediates												
1	2AP	-17.431	7.141	1.481	-74.436	-0.072	29.794	25.565	-32.667	24.818	55.874	51.965	16.333
2	2AP*	36.773	46.724	**49.904**	-43.573	59.400	66.802	**70.871**	17.558	87.370	92.202	**97.041**	85.938
3	2APqe	40.629	56.762	52.249	-41.936	66.755	77.456	75.648	22.861	93.995	98.566	99.678	90.601
	Enzymatic pathway												
4	D1	15.388	39.966	41.352	-106.854	38.580	58.428	60.527	-22.479	64.880	77.958	80.177	66.781
5	D2	-10.561	22.615	13.775	**-109.852**	4.591	40.390	33.802	**-48.200**	26.731	59.440	56.422	**30.310**
6	D3	33.655	47.391	42.839	-90.037	53.346	62.764	60.205	-9.404	79.336	82.734	83.125	79.486
7	D4	36.309	54.684	47.059	-92.084	60.025	72.552	67.666	-6.092	86.635	71.732	90.486	85.608
8	D5	39.123	52.428	55.737	-90.281	61.768	70.691	74.807	-5.238	87.248	91.141	94.557	85.722
9	D6	17.741	41.744	31.103	-104.878	39.556	57.051	50.309	-5.908	63.466	72.411	71.039	59.572
10	D7	-11.311	25.482	16.666	**-108.281**	4.698	42.627	36.352	**-46.753**	27.128	61.107	57.402	**28.397**
11	D8	35.010	50.540	46.310	-96.759	53.907	65.201	62.897	-14.847	78.207	84.581	84.057	75.203
12	D9	36.472	52.328	48.910	-96.318	59.149	70.003	68.486	-10.766	83.819	86.873	89.126	78.984
13	D10	37.870	51.532	49.163	-90.484	60.674	68.895	68.277	-6.589	84.684	87.875	89.017	81.701
	Non-enzymatic pathway												
14	D1'	49.739	61.771	56.314	-86.567	71.139	77.774	68.723	-2.120	93.579	94.794	97.113	86.860
15	D3'	68.794	68.297	63.722	-82.331	86.455	82.723	79.801	-2.200	107.455	100.673	99.551	87.080
16	XAN		63.213				81.117				98.097		

Bold: anionic forms were optimized in water solution.

**Fig 4:**
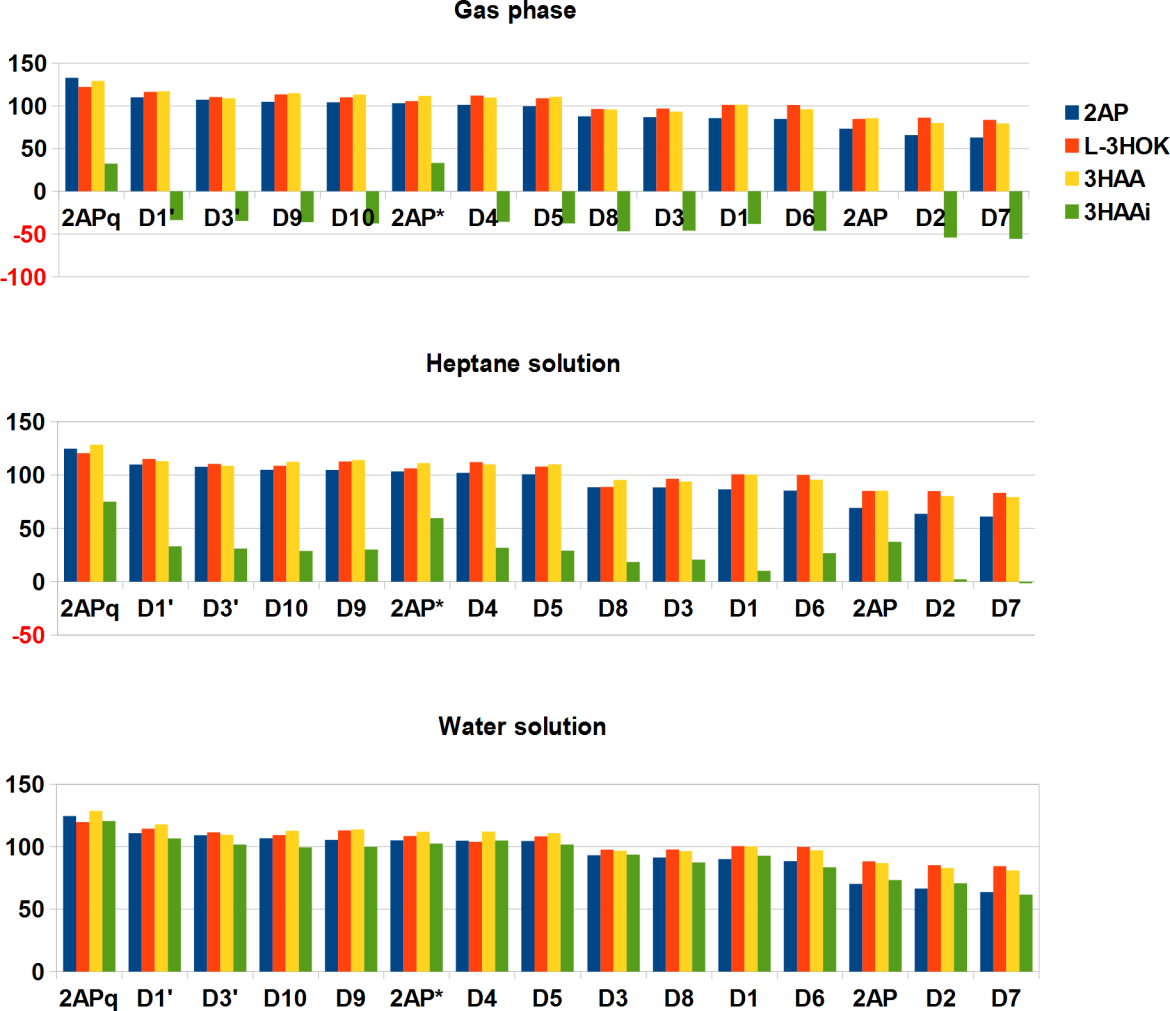
Electronegativity of R-2AP dimerization products. All values are in kcal/mol. Here and after: legends are given for corresponding R-2AP monomers and their dimerization products.

To reveal the summary trend, we have calculated the Mulliken electronegativity (χ) which is a half-sum of IP and EA (Fig 4). Again, we see that 3HAAi-D have a lower χ compared to the uncharged 3HAA-D. 2AP-D is slightly less electronegative compared to L-3HOK-D, as well as to 3HAA-D. R-2APq and D7 have the highest and the lowest χ, respectively. The trends of χ change are similar to that for EA: 1. χ is higher for the oxidized forms of compounds compared to their restored forms; 2. χ is higher for tautomers with disrupted aromaticity compared to those with the aromatic ring. For the compounds containing 2AP moiety, χ decreases in the raw: 2AP–D2 –D7, same as for IP values.

Summing up, L-3HOK-D and 3HAA-D are slightly better electron acceptors and worse electron donors compared to 2AP-D. However, as 3HAA derivatives are mainly in ionized form at physiological pH, their electronegativity should be more like as that for 2AP-D. Among all the studied compounds, 2-aminophenolic derivatives (D7, D2, 2AP) are the worst electron acceptors, while D7, D2 and their radical forms are the best electron donors. Hence, they should be the most powerful electron-donating antioxidants among all R-2AP derivatives. However, in case of their redox potential being too low, they are able to convert molecular oxygen to toxic ROS. Most 3HAAi–D can reduce O_2_ to superoxide anion radical O_2_*^-^ in the gas phase and in heptane solution (see Table 3). However, this reaction is thermodynamically forbidden in water solution, even for the most powerful electron donor 3HAAi-D7 (IP 94.892 kcal/mol). Possibly, O_2_*^-^ can be formed in the presence of metal ions such as Cu^2+^, which are known to increase 3HAAi electron-donating power [33]. In such case, 3HAAi-D7/ D8 are the most probable O_2_*^-^ generators.

For different R-2AP derivatives, there is a strong Pearson correlation among their BDG, IP, EA, or χ values in water solution (Tables II-IV in S2 Table). In the gas phase, these values for 3HAAi D do not correlate with those for the other dimers, because of the high influence of the charged COO^-^ groups on the redox properties and H-atom donating power. This effect is extinguished to some part in heptane. Here, R-2AP-D and especially 3HAAi-D demonstrate higher ability to produce toxic ROS compared to that *in aqua*. However, R-2AP derivatives should have different solubility in the lipid phase, which affects their ability to penetrate through the hydrophobic lipid bilayers. The additional factors should be considered, such as the affinity to enzyme and metal ions within the cell, as well as to specific membrane transporters, which in complex determine R-2AP-D availability for interaction with O_2_ and membrane lipids.

### Kinetics of molecular oxygen interaction with R-2AP non-enzymatically formed dimers

**Fig 5:**
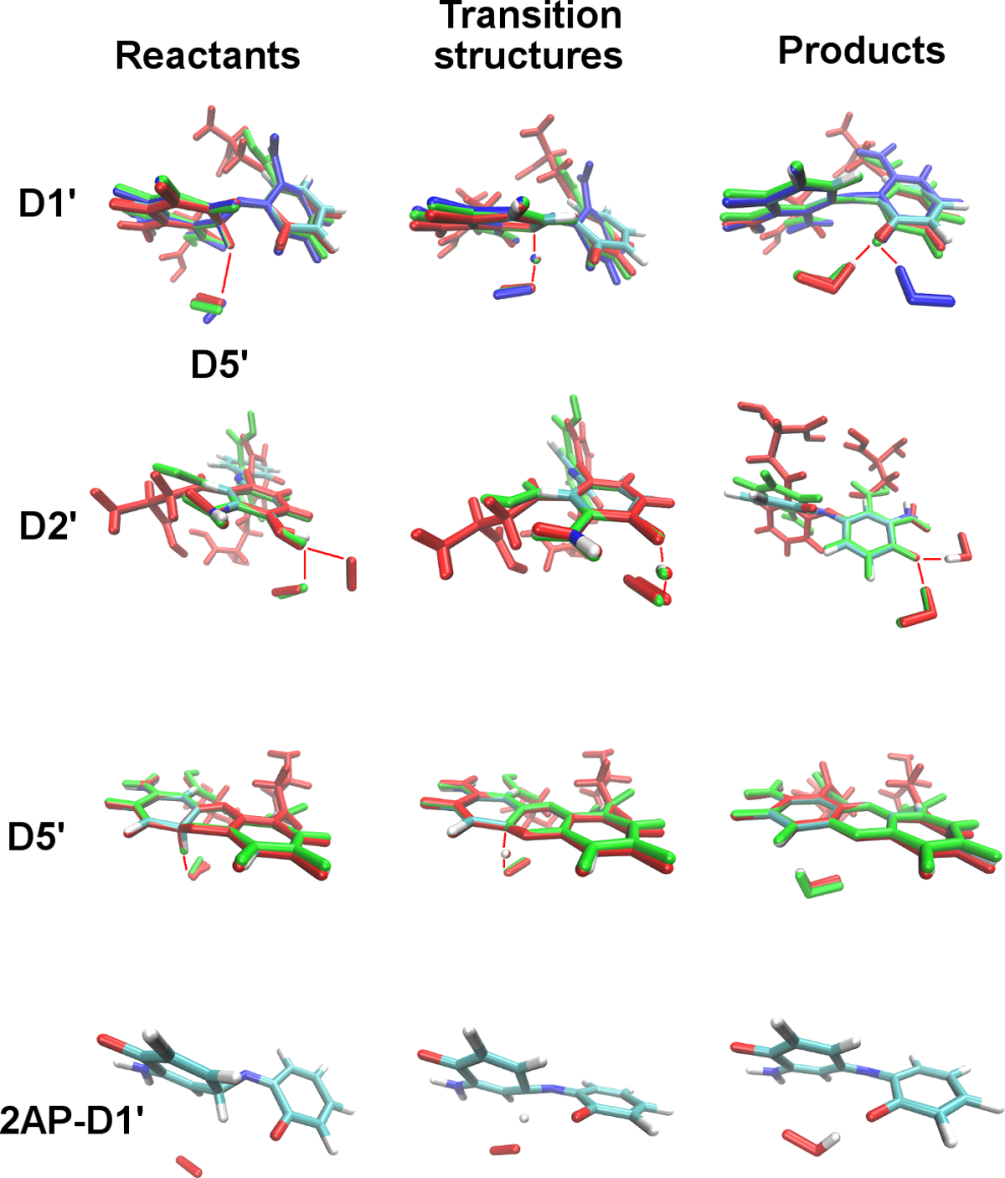
Kinetic of O_2_ interaction with non-enzymatically formed R-2AP dimers. Color scheme: 2AP, atoms: C–cyan, O–red, N–blue, H–white; kynurenines: red–L-3HOK, green– 3HAA, blue– 3HAAi.

As it has been said before, C-H homolytic bond dissociation in D1' (stage N16) and D5' (N21) is more energetically favorable compared to O-H bond dissociation in D2' (N19). However, N19 conversion due to D2' interaction with O_2_ may occur faster than that for N16 and N21. To check this hypothesis, we have calculated the reaction rates for the corresponding stages, as well as for the reverse stages (Fig 5 and Table 6). For 3HAAi, only D1'-O_2_ transition state (TS) occurred to be stable with our computational approach.

**Table 6:** Thermodynamic and kinetic parameters of R-2AP dimers H-atom donation to O_2_.

	Gas					Heptane				Water			
Form	ν_i_	ΔG_TS-R_	ΔG_TS-P_	k(T)_TS-R_	k(T)_TS-P_	ΔG_TS-R_	ΔG_TS-P_	k(T)_TS-R_	k(T)_TS-P_	ΔG_TS-R_	ΔG_TS-P_	k(T)_TS-R_	k(T)_TS-P_
2AP													
D1'	1702.2	21.684	28.246	7.33E-002	1.13E-006	20.512	26.957	5.30E-001	1.00E-005	19.152	26.707	5.26E+000	1.52E-005
D2'	1426.7	15.754	9.478	1.27E+003	5.07E+007	15.270	9.095	2.88E+003	9.68E+007	13.260	7.645	8.57E+004	1.12E+009
D5'	2297.8	33.439	21.308	2.85E-010	2.22E-001	32.729	21.308	9.43E-010	2.22E-001	30.819	22.218	2.37E-008	4.78E-002
L-3HOK													
D1'	1841.9	26.384	28.168	2.96E-005	1.46E-006	25.808	27.555	7.83E-005	4.10E-006	23.568	27.695	3.43E-003	3.24E-006
D2'	1385.9	15.373	16.224	2.33E+003	5.54E+002	14.325	17.961	1.37E+004	2.95E+001	13.665	15.141	4.16E+004	3.45E+003
D5'	2089.6	34.355	21.234	5.19E-011	2.16E-001	32.479	20.242	1.23E-009	1.15E+000	31.459	20.112	6.88E-009	1.43E+000
3HAA													
D1'	1883.6	27.034	29.501	1.02E-005	1.59E-007	26.164	28.827	4.44E-005	4.96E-007	24.764	28.937	4.72E-004	4.12E-007
D2'	1424.2	15.283	8.012	2.81E+003	6.01E+008	14.452	8.240	1.14E+004	4.09E+008	13.272	7.890	8.37E+004	7.38E+008
D5'	2071.5	33.305	19.130	3.01E-010	7.41E+000	32.785	19.092	7.24E-010	7.89E+000	31.235	20.242	9.90E-009	1.13E+000
3HAAi													
D1'	1706.1	20.483	35.363	5.59E-001	6.91E-012	20.682	33.797	3.99E-001	9.71E-011	19.932	30.887	1.42E+000	1.32E-008

Energies are in kcal/mol. ν_i_ values are in cm^-1^. k(T) values are in M^-1^s^-1^.

N16 is significantly slower than N19. At the same time, N16 is virtually irreversible, while the reverse reaction is significantly faster for N19 stage, except for L-3HOK-D3'. In N16, ΔG is lower for the products of reaction, whereas in N19, it is lower for the reactants, making D2'-O_2_ complex more stable compared to D3'-HO_2_*.Thus, D1' can be directly converted to D3' via H-atom abstraction by O_2_, making N16 reaction a potential source of HO_2_*. In N16, HO_2_* interacts with C = O group of the second aromatic ring, in N19, it remains in complex with C-O* group. This may prevent HO_2_* diffusion into the surrounding medium, partially decreasing the reaction rate.

N21 is dramatically slow, the reverse reaction being several orders of magnitude faster. The spin value is 1.5 for D5'–O_2_ complexes (including reactants, TS and products), corresponding to three unpaired electrons in the orbitals. Hence, N21 reaction is spin-forbidden, as O_2_ in the ground state has a spin value 1. Thus, to be oxidized, D5' should firstly be converted to D8: H-atom migrates from C- to N-atom, thereafter being abstracted by molecular oxygen.

In N16, the reactants, TS and products are in similar conformations for all 2AP derivatives, except for 3HAAi-D3'–HO_2_* where HO_2_* is away from the first aromatic ring. Logs of the reaction rates in gas, heptane and water are highly correlated (Table V in S2 Table). The direct reaction rate is higher for 2AP-D1' compared to 3HAA-D1' and L-3HOK-D1'. 3HAAi-D1' reacts most quickly with O_2_ except in water solution, the reverse N16 reaction for 3HAAi-D1' is very slow. In N19, O_2_ and HO_2_* positions differ for the studied complexes, albeit their TS geometries are quite similar. However, their reaction barriers and rates are approximately the same, except for L-3HOK-D2' where the products–reactants ΔG is negative, possibly due to some relaxation of steric strain within D2' structure. In N21, the conformations are the same for all the complexes. However, as it has been aforementioned, this reaction in unlikely to occur.

### Modeling Drosophila PHS structure in complex with R-2AP

**Fig 6:**
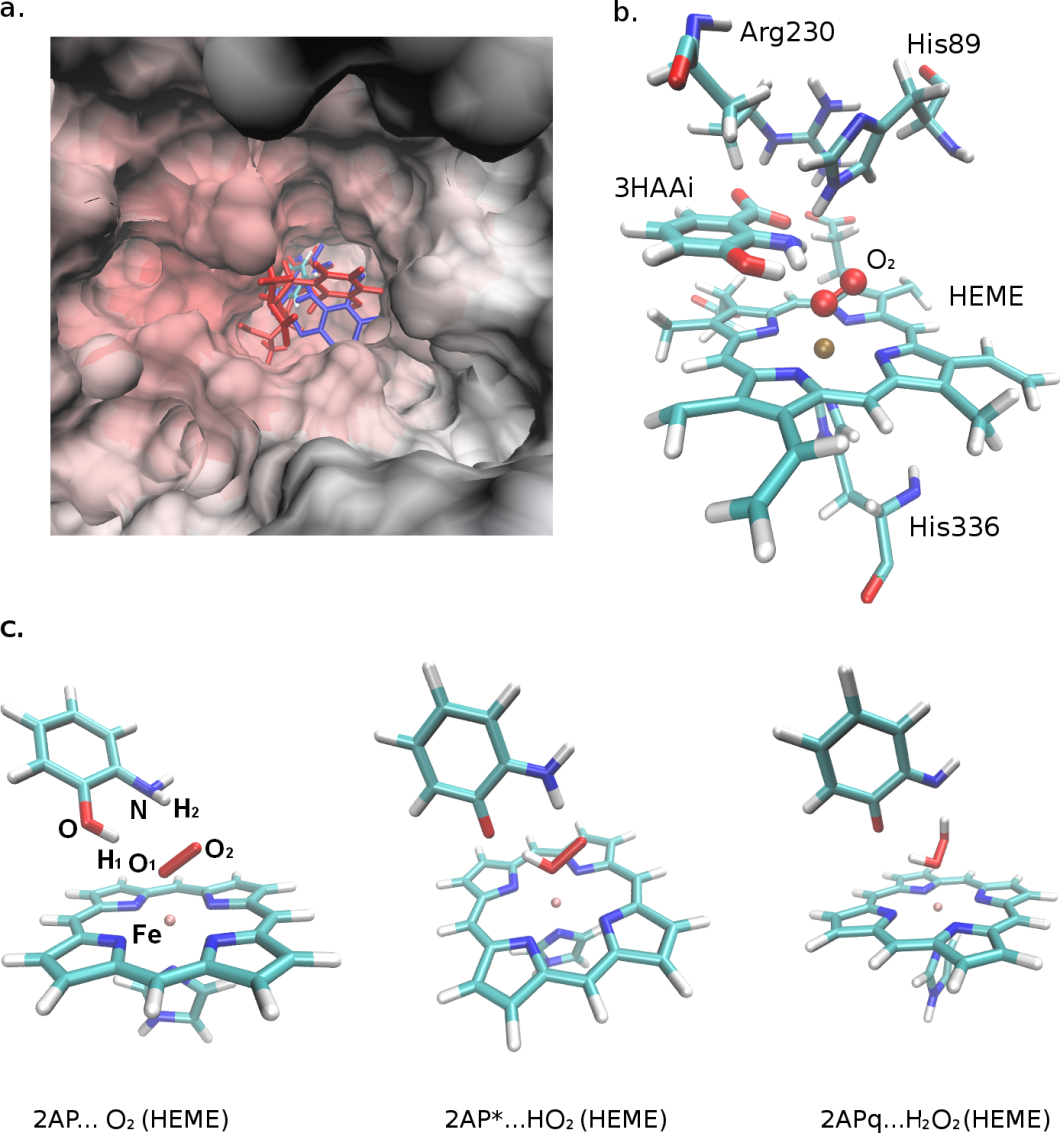
Drosophila phenoxazinone synthase (PHS) in complex with R-2AP. (a) PHS in complex with R-2AP; color scheme: 2AP, atoms: C–cyan, O–red, N–blue, H–white; kynurenines: red–L-3HOK, blue– 3HAAi. (b) PHS active site containing ferrous heme in complex with 3HAAi. (c) 2AP and products of its oxidation in complex with heme.

In Drosophila PHS, the putative catalytic site is similar to that in lactoperoxidase containing heme-binding residues, as well as the axial His residue. We can assume that PHS heme group in complex with O_2_ is in ferric form, similar to that in globins and TDO. Upon 2AP binding to PHS active site, its hydroxyl and amine hydrogens should be oriented towards O_2_. Automatic docking of R-2AP derivatives to PHS with oxo heme in the active site generated several structures, some of which meets the above criteria (Fig 6A and 6B). The binding pocket is wide enough for R-2AP and R-2AP-D2 to contact with O_2_ by their 2AP moiety. For R-2AP-D7 with a plane tricyclic group, there are no docked structures with such contacts to O_2_. The only exception is 3HAAi-D7 where OH and NH_2_ groups are oriented in the opposite way to that in 3HAAi-D2. Here, there are no interactions between enzyme and COO^-^ groups, and the total binding energy is relatively low. These data correspond to the experimental fact that PHS does not catalyze the last two H-atoms abstraction from R-2AP-D7.

The aromatic group of the substrate is nearly parallel to the heme plane, being ~3.2–3.5 Å above the pirrolic ring of heme. For 3HAAi, L-3HOKzi and their D2 derivatives, the PHS-substrate complex is stabilized by the ionic bond of COO^-^ group and Arg^230^. For L-3HOKzi and L-3HOKzi-D2, there is an ionic bond between αNH_3_^+^ and the heme COO^-^ group. His^89^ is above O_2_ and R-2AP aromatic NH_2_ group, being able to contact with them. The axial His^336^ is under the heme group (Fig 6B). The average distance between R-2AP O/N atoms and corresponding O atom of O_2_ is ~1.8/2.1 Å. The binding energy is minimal for 2AP and maximal for L-3HOKzi (Table 7). The second OH group in 2AP-D2 contacts with Thr^94^ C = O, and the second αNH_3_^+^ group in L-3HOKzi-D2 contacts with Asp^229^, that must additionally stabilize the enzyme-substrate complexes.

**Table 7:** Parameters of R-2AP-D complexes with Drosophila phenoxazinone synthase (PHS).

Substrate	Free energy (kcal/mol)	Number in cluster	Rank	OH_1_‥ O distance (Å)	NH_2_‥ O distance (Å)	O-H_1_-O angle (°)	N-H_2_-O angle (°)
2AP	-4.82	5	3	1.64	2.02	149.59	150.36
3HAAi	-5.23	10	1	1.93	2.03	158.36	133.14
L-3HOKzi	-7.82	9	1	1.78	2.25	141.95	133.05
D2-2AP	-6.51	2	3	1.67	1.82	150.52	144.01
D2-3HAAi	-5.48	4	1	1.85	2.27	145.26	139.15
D2-L-3HOKzi	-7.47	1	1	1.97	2.62	139.97	169.28
D7-3HAAi	-4.84	1	4	1.89	1.62	123.43	144.45

Number in cluster–the number of structures (10 is maximal) with similar orientation within PHS catalytic site; rank–the rank of cluster arranged by the free energy of substrate binding.

**Fig 7:**
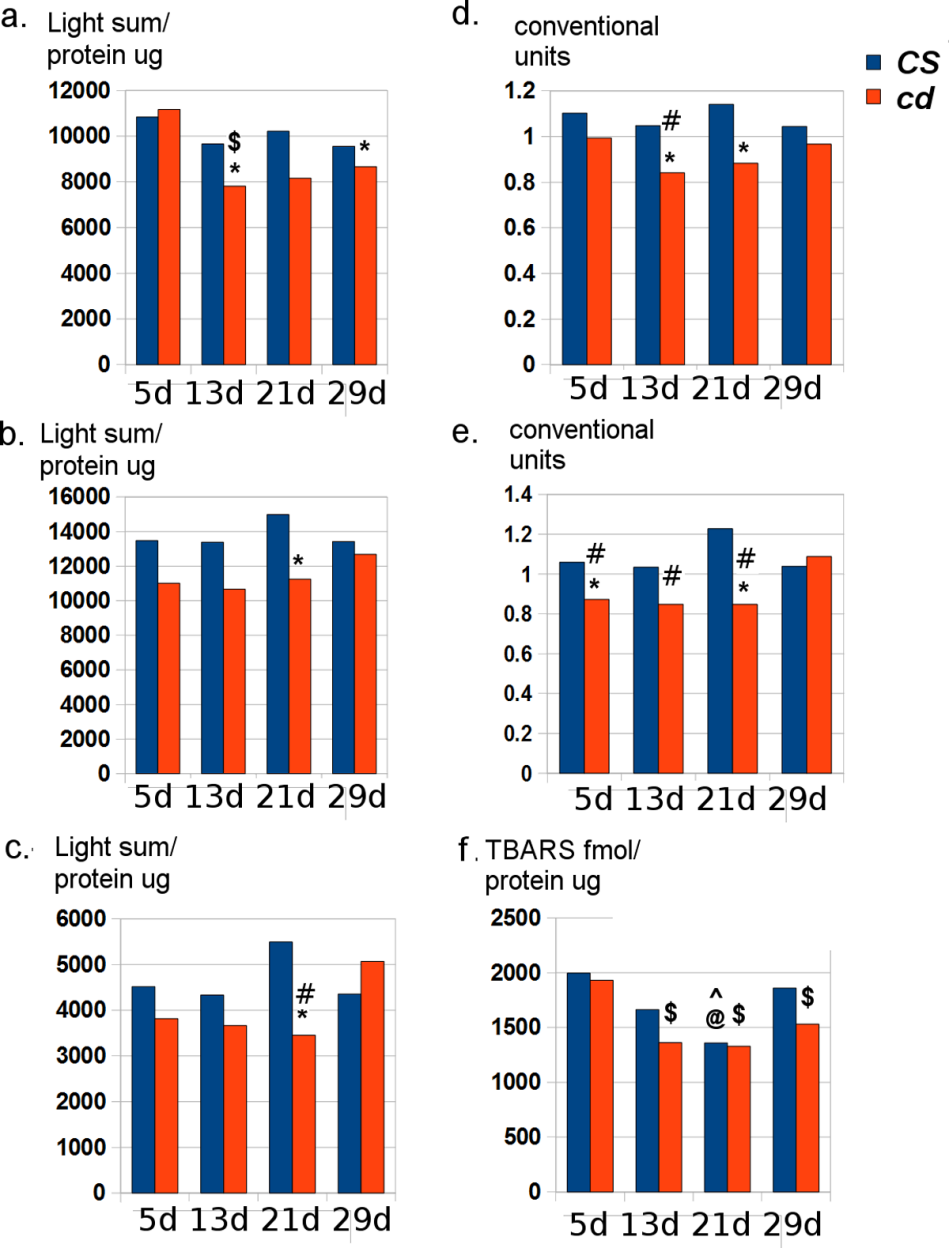
Total antioxidant capacity (TAC) and lipid peroxidation (LP) in Drosophila heads. a.—e. TAC: a. 25°C, 2000g; b. 22°C, 2000g; c. 22°C, 10000g; d.–normalized for a. and b.; e. normalized for b. and c; f. LP (22°C, 10000g).* cd vs CS, *^ CS vs CS 5d; $ cd vs cd 5d*, *@ CS vs CS 29d*, *# cd vs cd 29d* (two-sided randomization test; p <0.05).

O_2_ in the ground state in complex with porphyrin group of heme and imidazole group of the axial His has the total spin value 0, corresponding to quantum mixture of two configurations: Fe(II)-O_2_ (ferrous heme) and Fe(III)-O_2_^–^ (ferric heme) [34]. Fe(II) and Fe(III) ions significantly enhance the rate of DOP H-atom abstraction by O_2_ and H_2_O_2_ formation [35,36]. To check the influence of ferrous iron on 2AP H-atom abstraction by O_2_, we calculated the free energies of 2AP interaction with: a. O_2_ (triplet state); b. O_2_ in complex with FeCl_2_ (singlet and triplet states); c. O_2_ in complex with porphyrin group of heme and the axial imidazole (singlet state) (Fig 7C and Table 8). In the absence of iron, H-atom abstractions from 2AP leading to HO_2_* and H_2_O_2_ is thermodynamically unfavorable. In the presence of FeCl_2_, 2AP conversion to HO_2_* is favorable for the complex in triplet state. 2AP conversion to H_2_O_2_ remains unfavorable, but ΔG significantly decreases. Thus, inorganic iron can facilitate two-step 2AP oxidation to H_2_O_2_. For 2AP complex with Fe-oxo-heme-imidazole in singlet form, all oxidation steps are only slightly endothermic, which makes PHS-2AP complex a potential source of ROS, including HO_2_* and H_2_O_2_. Both inorganic and heme iron induce the partial negative charges on O_2_ atoms that may facilitate H-atom abstraction.

**Table 8:** 2AP interaction with the oxygen forms (level I).

Reactants and products	ΔG	Spin	Mulliken charges							
			C(O)	O	H_1_(O)	O_1_	O_2_ (Fe)	Fe	H_2_(N)	N
2AP +O_2_ →	13.006	1	+0.34	-0.66	+0.42	+0.01	0.00		+0.33	-0.83
2AP* + HO_2_* →	36.329	1	+0.40	-0.59	+0.48	-0.30	-0.21		+0.40	-0.80
2APq^#^ + H_2_O_2_		1	+0.42	-0.51	+0.45	-0.43	-0.43		+0.45	+0.31
2AP +O_2_ (FeCl_2_) →	8.391	0 ^$^	+0.31	-0.65	+0.46	-0.31	-0.31	+0.92	+0.38	-0.82
2AP* + HO_2_*(FeCl_2_) →	4.695	0 ^$^	+0.43	-0.55	+0.50	-0.46	-0.37	+0.80	+0.44	-0.77
2APq + H_2_O_2_ (FeCl_2_)		0	+0.46	-0.48	+0.47	-0.39	-0.49	+0.73	+0.51	-0.66
2AP +O_2_ (FeCl_2_) →	-0.635	1	+0.38	-0.61	+0.50	-0.44	-0.40	+0.90	+0.46	-0.79
2AP* + HO_2_*(FeCl_2_) →	6.440	1	+0.41	-0.61	+0.52	-0.46	-0.35	+0.89	+0.41	-0.80
2APq + H_2_O_2_ (FeCl_2_)		1 ^$^	+0.45	-0.50	+0.46	-0.39	-0.51	+0.74	+0.52	-0.68
2AP +O_2_ (Heme) →	1.037	0	+0.31	-0.68	+0.47	-0.25	-0.19	+1.68	+0.35	-0.84
2AP* + HO_2_*(Heme) →	6.822	0 ^^^	+0.40	-0.59	+0.52	-0.52	-0.38	+1.69	+0.42	-0.80
2APq + H_2_O_2_ (Heme)		0	+0.44	-0.49	+0.51	-0.52	-0.42	+1.55	+0.46	-0.62

“Heme” stands for porphyrin group in complex with Fe^2+^ and axial imidazole. ΔG values (kcal/mol) are given for the corresponding reaction shown by arror. For 2AP complexes with molecular oxygen and FeCl_2_:

$ spin state corresponding to structure with maximal negative energy.

^ spin contamination: spin-squared is ~ 1 with unrestricted DFT, the structure is unstable with restricted open-shell DFT, being spontaneously converted to [2AP+O_2_ (Heme)];

# optimized in water.

### Age-dependent total antioxidant capacity and lipid peroxidation in Drosophila kynurenine mutant *cd*

To estimate the correlation between L-3HOK accumulation and oxidative stress development in *D*. *melanogaster*, we measured TAC and LP in male heads at different stages of adult life. *cd* strain (2.9X L-3HOK accumulation [24,27]) was compared to the wild-type strain *Canton-S (CS)* (Fig 7). To check the possible influence of the fly keeping temperature and the quality of samples purification (centrifugation strength) on TAC, we performed several experiments varying these parameters (a.–e.). TAC is decreased in *cd* relative to *CS* in 5 day-old flies kept at 22°C (e.) and 29-day-old flies kept at 25°C (a.). There is a significant relative TAC decrease in *cd* at the 13^th^ day (a., d.) and the 21^th^ day (b.–e.). We do not see any significant age-dependent changes in *CS* TAC, while *cd* shows TAC decrease at the 5^th^–13^th^ day interval (a.) or TAC increase from midlife to the 29^th^ day (c.–e.). Thus, a strong tendency to TAC decrease in *cd* is observed, being the most reliable for middle-aged (13–21-day-old) flies. LP is somewhat lower in *cd* than in *CS*, however, there are no statistically significant LP differences between two strains (f.). At the same time, LP decreases in *cd* after the 5^th^ day, remaining comparatively low during the total studied period, whereas in *CS* LP decrease is observed only at the 21^th^ day. Thus, two effects can be observed simultaneously: 1. the age-dependent decrease in *cd* TAC, probably corresponding to the toxic effects of L-3HOK accumulation in fly heads; 2. the age-dependent increase in *cd* ability to inhibit lipid peroxidation, possibly connected to the peroxy radical scavenging activity of L-3HOK and its dimers.

## Discussion

Kynurenines, the products of tryptophan catabolism, are compounds with a broad spectrum of neurobiological activities, involved in the development of various neuropathologies, such as HD, PD, AD, schizophrenia, depression, dementia-like disorders, and suicide [2,28,37]. Most of kynurenines are able to modulate cell receptors, but there are no known specific targets for them. For all the aromatic amino acids, their major catabolism pathway is biochemically separated from that leading to neuromediator production, which makes it possible to be tightly regulated in organism. The neuromediator synthesis includes the stage of decarboxylation: dopamine (DOP), the first catecholamine, is formed from dioxyphenylalanine (DOPA), histamine–from histidine, and serotonine–from 5-hydroxytryptophan. Some kynurenines, like KYN and L-3HOK, can be decarboxylated in liver and brain to kynuramines, a specific class of biogenic amines with rather poorly studied biological effects. Kynuramine itself competes with tryptamin receptor in the rat brain, also being an antagonist of α1-adrenergic receptor, while the melatonin products, 5-methoxylated kynuramines, are radical scavengers [38].

The main physiological function of kynurenines is to regulate the organism response to adverse environmental effects, such as infection or stress, providing defensive reactions on molecular and behavioral levels. While the hepar IDO is constutively active, being responsible for basic tryptophan catabolism, the brain IDO and some other KP enzymes are activated by different harmful agents like bacterial and viral products, as well as inflammatory cytokines [2,39,40]. L-3HOK is mainly produced by microglia, whereas KYNA is formed in astrocytes. Kynyrenine 3-monooxygenase is one of the key enzyme affecting the balance of neurotoxic and neuroprotective kynurenines, shifting KP from predominant KYNA production towards the generation of L-3HOK and QUIN, responsible for neurodegeneration and depression [41].

The neurotoxic effects of hydroxykynyrenines are caused by ROS hyperproduction, while QUIN is an excitotoxin activating NMDAR. QUIN is not synthesized in Drosophila. This makes *cd*, a Drosophila mutant with enzymatically disrupted XAN synthesis, a useful model to study specific effects of L-3HOK accumulation on neuropathology development [28]. 5-day-old *cd* male demonstrate a middle-term memory decrease after heat-shock–an effect prevented by adding phenolic antioxidants in the food media of developing flies [42]. Nowadays, there is a renaissance of interest in science to chemistry and physiology of ommochromes, the invertebrate pigments, including Drosophila brown pigments XAN and DXAN produced by L-3HOK dimerization, which also participate in many biological processes, such as electron transport and free radical trapping [43]. In mammals, different proteins can catalyze R-2AP oxidative dimerization, such as hemoglobin and peroxidase, including heme or copper ions in their catalytic sites [21]. Thus, dimerization of hydroxykynurenines is involved in many physiological processes connected to ROS metabolism.

It is still not completely clear which ROS are directly generated during kynurenines' dimerization, as well as what is the role of enzymatic and non-enzymatic pathways in their production. H_2_O_2_ and iron play an important role in 3HOK-induced cytotoxicity [44]. H_2_O_2_ seems to be the chief product of non-enzymatic 3HOK oxidation, as catalase but not superoxide dismutase blocks 3HOK-induced cell death [45]. H_2_O_2_ then generates highly toxic hydroxyl radicals that damage nervous cells [16]. However, H_2_O_2_ also can be formed due to O_2_*^-^ disproportionation [36]. 3HOK and 3HAA generate H_2_O_2_ in a Cu(II)-dependent manner, likely through anilino or phenoxyl radicals formation [46]. 3HOK adducts with lens protein are also generated upon the ultraviolet illumination, probably contributing to age-related development of cataract [47]. These processes are difficult to study experimentally, as most ROS are very active and easily convert to each other. The computational approach may be useful to reveal the mechanism underlying kynurenines’ dimerization leading to ROS formation.

In our studies, we were specially focused on the oxidative dimerization of hydroxykynurenines L-3HOK and 3HAA, as well as their structural precursor 2AP. The dimerization pathways, including both enzymatic and putative non-enzymatic stages, were computationally studied, considering the free energies of conversion of intermediates and their electron donation/acceptor power. For simplicity, the process of H-atom abstraction was modeled as a proton-coupled electron transfer (PCET) without separation of a proton and an electron migration stages.

In general, both H-atom and electron dissociation energies decrease as the dimers oxidation progresses, in agreement with [19]. However, BDG and IP values are minimal for the compounds with intact 2AP moiety, such as D7, being higher for the radical and/or quinoneimine forms. BDG is extremely low for C-H bond in non-enzymatically formed R-2APq conjugate D1', which dissociation seems to be energetically favorable due to the concomitant aromatic radical formation. The rate of C-H bond dissociation is lower compared to that for O-H bond in D1' tautomer D2'. However, D1' reaction with O_2_ is irreversible that makes it a potent source of hydroperoxyl radicals. The ionized COO^-^ groups in 3HAA dimers potentiate both electron- and H-atom donation, in agreement with [32]. 2AP moiety within the non-enzymatically formed dimers D7 and D2' can react with O_2_ generating H_2_O_2_ via the consecutive O-H and N-H bonds dissociation. In addition, H_2_O_2_ can be formed as a by-product of L-3HOK-D10 oxidation to XAN. The electron donation is more thermodynamically favorable in the lipid surrounding than *in aqua*.

The chemistry of kynurenines’ dimerization can be better understood on comparing them with catecholamines, the structurally related compounds with similar redox properties. Catecholamines are widely involved in multiple neurological processes in brain and periphery. Like L-3HOK, DOPA and DOP inhibit lipid peroxidation in complex with α-tocopherol at low pH, becoming pro-oxidants at pH 8.0 due to the interaction of semiquinone anion radical with O_2_ [48]. Catecholamines are susceptible to the oxidative dimerization to melanin producing ROS, toxically affecting the nervous system. Structures in brain accumulating catecholamines are prone to age-dependent or disease-induced degeneration, such as substantia nigra in PD. DOP interacts spontaneously with O_2_ in two steps, firstly producing O_2_*^-^ and *o*-semiquinone, which is then oxidized to 2APq analogue *o*-quinone. *o*-Quinone produces aminochrome and the eumelanin precursor 5,6-indolequinone, which both can form adducts with proteins involved in PD development, such as α-synuclein, parkin, actin, and tubulin [49,50]. The redox-active metals play a special role in the aforesaid processes: Fe(II) increases the rate of DOP oxidation to *o*-semiquinone by several orders of magnitude. O_2_*^-^ interaction with DOP is energetically unfavorable; however, O_2_*^-^ is much more powerful oxidant for *o*-semiquinone compared to molecular oxygen. H_2_O_2_ can be formed by O_2_*^-^ oxidation or disproportionation, as well as directly by leucoaminochrome or 5,6-dihydroxyindole oxidation [35,36].

For 2AP and its derivatives, L-3HOK and 3-HAA, we can suppose similar two-step oxidation by O_2_, leading firstly to semiquinoneimine (R-2AP*) and then to quinoneimine (R-2APq). O_2_ is triplet in the ground state, having two unpaired electrons. Hence, it is unable to react directly with most organic molecules with all paired electrons, except for those that can produce free radicals via one-electron transfer [51]. Upon binding O_2_, the ions of transition metals, such as iron and copper, easily change its spin state, making possible two-electron transfer to O_2_ from a substrate. One such activating compound is the heme group of globins, as well as of some peroxidases and dioxygenases. Fe-oxo-heme complex is formed in globins, which carry O_2_ in blood and muscle tissue. Similar complex is thought to catalyze the oxidation of L-tryptophan to N-formylkynurenine in the active site of TDO. Heme-containing enzymes reduce O_2_ using PCET mechanism, leading to formation of Fe(III)-OOH* heme complex (ferrix-hydroperoxy intermediate, also known as Compound 0). In peroxidases, this complex is formed upon H_2_O_2_ binding to ferric heme. Being protonated, it rapidly abstracts a water molecule, turning to Fe(IV) = O^+*^ heme (Compound I). After that, the active site should be restored by some appropriate enzyme [52,53]. Compound I is believed not to be formed in TDO active site [52]. Hence, the cleavage of O-O bond should occur after O_2_ binding to substrate.

L-3HOK, 3HAA and 2AP can bind to ferrous oxy-hemoglobin converting it to ferric met-hemoglobin, and vice versa [54,55]. Drosophila PHS seems to form similar complexes with R-2AP. In the case of two simultaneous H-atoms abstraction by O_2_, R-2APq and H_2_O_2_ would form and immediately dissociate from the active site. However, PHS is known to produce two water molecules upon 2AP enzymatic oxidation [21]. Hence, only one of two hydrogens, likely hydroxyl H-atom, should be transferred to O_2_ at the first stage. In the catalytic site of PHS, the oxidation seems to pass step-by-step without leaving unreduced oxygen forms from the enzyme to the extra medium. According to our data, both FeCl_2_ and ferrous heme significantly decrease the energies of two-step 2AP H-atoms abstraction by O_2_, making HO_2_* and H_2_O_2_ production energetically favorable or low-cost. HO_2_* protonation by some residue within PHS catalytic site probably assists O-O heterolysis in Compound 0, producing water and Compound I, likewise in cytochrome c peroxidase and horseradish peroxidase [56]. This prevents H_2_O_2_ formation by the enzyme, which therefore serves as a component of antioxidant system, prohibiting ROS formation at the first stages of R-2AP dimerization. At the same time, H_2_O_2_ formation seems to occur non-enzymatically upon R-2AP interaction with O_2_, especially in the presence of iron.

**Fig 8:**
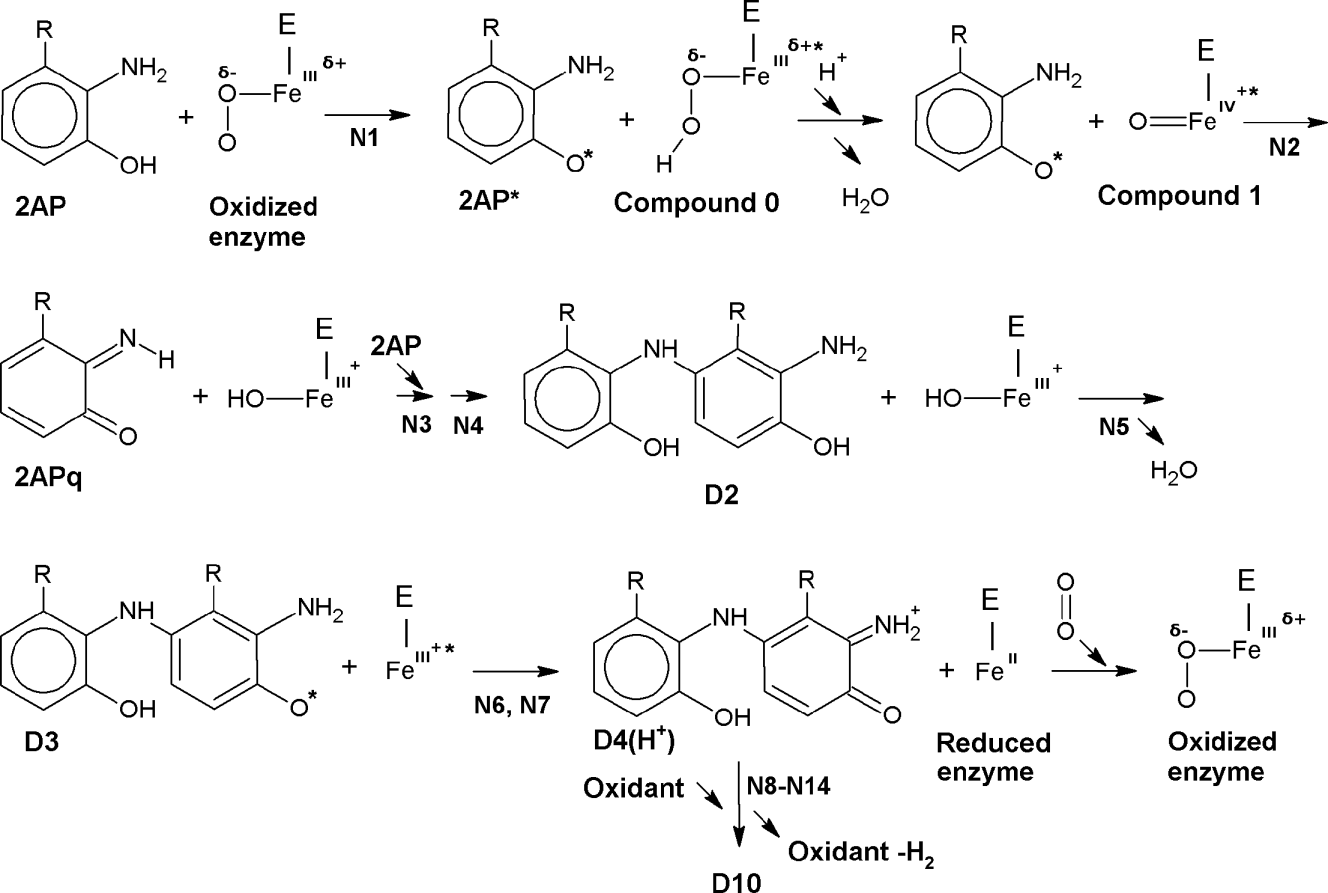
Hypothetic scheme of R-2AP and oxo-heme conversion in the catalytic site of Drosophila PHS.

The hypothetic scheme of the whole PHS cycle is shown in Fig 8.

Here, the first H-atom abstraction by O_2_ generates 2AP* and Compound 0. Being protonated, possibly by His^89^, Compound 0 abstracts a water molecule and turns to Compound I, which is then partly reduced by the amine H-atom, converting 2AP* to 2APq. Then another 2AP molecule attaches 2APq and restores its amine and hydroxyl groups (D2 formation). The second hydroxyl H-atom abstraction by D2 generates the second water molecule, reducing PHS active site to ferric form in one or several steps. The ferric form is then reduced to the initial ferrous form, possibly with an electron donated by D3. Hence the enzymatic stage of 2AP cyclization is ended with D4(H^+^), which is then undergoes a non-enzymatic cyclization to D10.

The previously mentioned scheme assumes that a substrate remains in the active site of PHS until the full reduction of the O_2_-heme complex. PHS thereby prevents formation of toxic non-reduced oxygen forms during 2AP and R-2AP oxidative dimerization. As it has been proposed earlier [19], 2AP moiety is regenerated after conjugation via tautomerization including two H-atom migration to the quinone group. According to our data, D2 has the lowest BDG among the enzymatically converted 2AP derivatives, and D2/D3 have the lowest IP, which makes them easy to donate H-atom and electron, respectively. The following non-enzymatic stages seem to be the main source of ROS, such as HO_2_* and O_2_*^-^. Thus, PHS prevents ROS formation at the first stages of kynurenines’ oxidation, which are greatly facilitated in presence of transition metals. Some other unknown antioxidant protection system should be involved at the further stages. In cell, PHS may be in complex with the other enzymes, which can assist R-2AP final oxidation or detoxify ROS, the by-products of XAN and CIN formation. Some oxidant or oxidants should participate at the final stages, specifically attaching the last two H-atoms. For example, XAN in Drosophila can catalyze its own formation, serving as H-atoms and/or electrons acceptor [29].

Little is known about the age-dependent changes of kynurenines’ level in adult Drosophila, as well as about the molecular mechanisms of L-3HOK accumulation in *cd*. An interesting fact experimentally shown by us is that TAC is decreased in *cd* heads compared to the wild type *CS*, being lowest at the 13^th^-21^th^ days of life, while LP is at the same level for both strains and specifically decreases in *cd* after the 5^th^ day. These two effects seem to partially offset each other, as they are not too pronounced and not stably observed for all fly ages in all experiments. Changing the temperature of flies keeping from 22 to 25°C accelerates flies ageing, which can move the age of major TAC differences between two strains from the 21^th^ to the 13^th^ day. TAC is almost equal for both strains at 29^th^ day when flies are kept at 22°C, remaining lower for *cd* at 25°C. Thus, high temperature may contribute to the oxidative stress development in old Drosophila. LP decreases in the aged *cd* and remains low throughout the studied period of life compared to 5-day-old flies. This might be caused by chronic accumulation of L-3HOK and/or L-3HOK-D, which antioxidant activity is primarily connected to H-atom donation power. The same accumulation would increase the possibility of HO_2_* and H_2_O_2_ formation, which in turn triggers the complex reactions leading to oxidative stress. The causal connection between L-3HOK accumulation in *cd* and TAC or LP decrease remains to be confirmed experimentally.

To summarize, we have computationally shown that H-atom and electron donation power of 2AP and hydroxykynurenines progressively increases upon their oxidative dimerization. Being regulated by PHS or the other enzymatic systems, this process does not lead to harmful effects associated with ROS hyperproduction. However, when the enzymatic activity is disturbed or L-3HOK level is too high, the same process begins to pass non-enzymatically, being accompanied by free radicals production, especially in the presence of transition metal ions. The total effect of L-3HOK accumulation seems to be noxious, though it can decrease some aspects of the oxidative stress, connected to lipid peroxidation. Thus, we can use two principle strategies to prevent the neurotoxicity caused by hydroxykynurenines’ self-oxidation: 1. To block the whole KP or some its stages in the affected tissue using synthetic inhibitors, as it had been proposed earlier [1,2]; 2. to elaborate the therapeutic approach to stimulate the activity of enzymes specifically converting kynurenines and ROS to non-active forms. Studying PHS function in Drosophila, its mechanisms of action, regulation, age-dependent activity changes, interaction with the other proteins, and the molecular nature of possible PHS damages in *cd* can help us in this.

## Materials and methods

### Initial structures for B3LYP geometry optimization

To facilitate the readability of dimer names in text, all of them were coded as following: “X-Dn”, where “X” is the monomer name (2AP, L-3HOK, 3HAA, 3HAAi, and R-2AP–for the last three monomers), “D” stands for “dimer” or “dimers” and “n” is the dimer number according to scheme of dimerization (see Figs 1 and 2).“D'n” indicates dimers converted via the non-enzymatic pathway. The reaction numbers are shown with the letter “N”. The enzymatic dimerization was modeled according to the schemes given in [19, 21], with the following modifications: 1. R-2AP quinoneimine monomers were modeled in the uncharged form; 2. two consecutive stages of H-atom abstraction were added after D2 (D3, D4) and D7 (D8, D9) (Fig 1). The non-enzymatic dimerization was modeled according to the scheme in [20]: 2APq → D1' → D3' → D10, with the addition of several hypothetical intermediates (Fig 2). The influence of hydrophobic (heptane) and aqueous surroundings on R-2AP dimerization energies, IP, and EA was studied.

The structures of 2-aminophenol (2AP), 2-aminophenoxazin-3-one (2APX), L-3HOK, 3HAA, XAN and DXAN were taken from the PubChem Compound database [57]. The structures of all dimerization intermediates were constructed on the base of PubChem structures using Vega ZZ 3.0.3 [58]. The initial energy minimization and systematic conformational search of low-energy geometry for all non-radical compounds was performed with the help of Avogadro [59] and Confab [60] using MMFF94 force field [61]. The carboxylic groups of 3HAA and its dimerization products are in ionized form at physiological pH (7.4), while L-3HOK and its dimers are mainly in zwitterionic form [62]. The ionized 3HAA (3HAAi) and 3HAAi-D were modeled with total charge -1 and -2, respectively, as well as their uncharged forms. L-3HOK zwitterions are not stable in the gas phase, and their optimization is hardly to converge in water solution. Hence, L-3HOK and L-3HOK-D were modeled in the neutral form. The initial geometries of radical structures (after H atom abstraction or electron addition/ abstraction) were set equal to that for the paternal closed shell structures. For quinoneimine structures, there are two possible conformers with N-H bond orientations: a. towards C = O bond, b. away from C = O bond. The B3LYP-optimized conformer a. had the lower energy compared to conformer b., except for 3HAAi and its derivatives. We performed all the calculations for the respective conformers with the lowest B3LYP energy. D1 and D6 have a chiral C atom. Since dimer conjugation is not stereospecific [19], all the calculations have been performed for a single optical isomer of D1 and D6, as well as their derivatives D1' and D5'/D6'.

### Quantum chemical geometry optimization of local minima and single-point energy calculations

All quantum chemical calculations were performed using Firefly 8.1.0 partially based on the GAMESS (US) [63] source code. Firefly 8.1.0 was kindly provided by Alex A. Granovsky [64]. For 2AP complexes with ferrous forms, the geometries were fully optimized using density functional theory (DFT) at B3LYP/6-31G(d) level (I). For the other compounds, the optimization was performed at B3LYP/6-311G(d,p) level (II) [65–67]. B3LYP1 version of B3LYP was used. All closed shell molecules were calculated in a singlet state, using restricted DFT method. Doublet state was used for radical structures and triplet–for D6' and complexes with molecular oxygen, using the unrestricted DFT method. All R-2AP-D radicals were optimized using the corresponding closed shell form as the initial conformation. For the most complexes with ferrous forms, the restricted open-shell DFT method was used. The symmetry point group was set as C1 for all compounds. Hessian matrix, vibrational frequencies and thermal corrections to the total energy at 298.15 K were calculated using the same method as for the geometry optimization. The nature of all stationary points was determined by evaluating the vibrational frequencies. Several of the ion-radicals, mainly those with the ionized carboxylic groups, are not stable in the gas phase, and their optimization was performed in water solution using DPCM model [68], without cavitation, dispersion and repulsion free energies.

For the optimized structures, single point energy calculations were performed at B3LYP/6-311+(O)+G(d) level (III). The calculations were performed both in the gas phase and in heptane or water solution at 298.15 K using DPCM. Due to the bad SCF convergence, p polarization functions at H atoms were omitted, and the diffuse functions were added only to O and H atoms. To check whether it affects the relative order for X-H bond dissociation energies, we performed the linear regression analysis for 36 X-H dissociation energy values calculated at level II and III in the gas phase, where X is O, N or C-atoms: energy (III) = 0.977 *x* energy (II)-0.916. The energy difference are maximal for O-H dissociation energies (-4.476±0.787 kcal/mol). Hence, at level III, O-H bond dissociation energies are somewhat decreased relative to those for N-H and C-H bonds. This may be caused by the greater opportunity for the unpaired electron to be delocalized on O-atom diffuse orbital. For proton, G_FREE_ was set to be -6.28 kcal/mol in the gas phase and -272.18 kcal/mol in water solution, as in [69]. In literature there are no data on proton G_FREE_ in heptane. Hence, we calculated the summary ΔG for the reaction pairs N7, N8 and N13, N14, including the sequential protonation and deprotonation of R-2AP-D.

### Free energies calculation

Gibbs free energy was obtained from the vibrational frequency calculations at 298.15 K, using unscaled frequencies:

$$G=E_{T}+G_{CORR}$$ (2)

where G is Gibbs free energy, E_T_ is a total energy of the optimized molecule (for the gas phase) or a total free energy in solution (for heptane and water solution) calculated at level III, G_CORR_ is a thermal correction to G calculated at level II.

The free energy change (ΔG) was calculated as follows:

$$\Delta G=G_{RAD}+G_{H}–G_{W}\left(\text{H-atom dissociation}\right)$$ (3)

$$\Delta G=G_{DIM}-G_{M1}–G_{M2}\text{(dimerization energy)}$$ (4)

where DIM is dimer, H is hydrogen, M1 is monomer 1, M2 is monomer 2, RAD is radical, W is the whole molecule (before H abstraction).

Ionization potential (IP) and electron affinity (EA) were calculated as follows:

$$IP=G_{CAT}–G_{W}$$ (5)

$$EA=G_{W}–G_{AN}$$ (6)

where AN is anion-radical (one-electron addition), CAT is cation-radical (one-electron abstraction).

The Mulliken electronegativity (χ) was calculated as follows:

χ=(IP+EA)/2 (7)

### Reaction rate calculations

For complex with molecular oxygen, transition states (TS) and corresponding local minima were optimized at level II. Intrinsic reaction coordinates (IRC) calculations [70] were performed for all TS species at the same level to confirm that anticipated reactants (R) and products (P) are connected to TS.

ΔG is corrected reaction activation energy:

$$\Delta G_{\#R}=G_{TS}-G_{R}$$ (8)

$$\Delta G_{\#P}=G_{TS}-G_{P}$$ (9)

$$\Delta G_{P-R}=G_{P}–G_{R}$$ (10)

where G_TS,_ G_R_ and G_P_ are free energies of transition state, reactants and products of reaction; ΔG_#R_ and ΔG_#P_ are reaction barriers for reactants and products; ΔG_P-R_ is the free energy change for reactants conversion to products, respectively. The rate of reaction (M^-1^s^-1^) between antioxidant and radical was calculated as in [32,71] using conventional TS theory:

$$k\left(T\right)=I\;x\;\left(k_{B}T/h\right)\;x\;[exp(-\Delta G_{\#}/RT)]\;x\;24.3\;x\;A\left(T\right)$$ (11)

where I is the reaction pathway degeneracy (1 for the all compounds), k_B_ is Boltzmann's constant, h is Planck's constant, ΔG_#_ is ΔG_#R_ or ΔG_#P_, 24.3 is a multiplier used to convert the units from 1 atmosphere standard state to 1 M standard state, and A(T) is a temperature-dependent factor corresponding to quantum mechanical tunneling, approximated by the Wigner method [72]:

$$A\left(T\right)=1+\left(1/24\right)\;x\;{\left(1.44\nu_{i}/T\right)}^{2}$$ (12)

where ν_i_ is the imaginary frequency (cm^-1^) whose vibrational motion determines the direction of the reaction.

Atom coordinates of the optimized structures are given in S1 Dataset. G and G_CORR_ values calculated at level II for ΔG, IP, EA, and k(T) are given in S3 Table.

For 2AP, L-3HOK and 3HAA, ΔG, IP and EA values calculated at level II and III in the gas phase are highly correlated (R^2^ > 0.99; Table VI in S2 Table). The same is true for 3HAAi ΔG (II) and (III). Hence, both methods give the same relative order of energy values. R^2^ is ~0.97 for 3HAAi IP (II–III) and ~0.77 for 3HAAi EA (II–III). The correlation decrease is possibly connected to facts that many 3HAAi forms are unstable in the gas phase and were optimized in water solution, but their single-point energies are given for the gas phase. Solvation effects significantly affect the relative order of IP and EA values, as it were shown here (Figs 4 and S2 and S3). For log k(T), there is a strong correlation between values calculated at level II and level III in gas, heptane, and water (Table VI in S2 Table).

For IP and EA, the average G_CORR_ is rather small, being 0.241±0.415 and 1.949±0.458 kcal/mol, respectively (n = 61). For X-H bond dissociation, the average G_CORR_ is -13.391±0.477 kcal/mol, and the average thermal enthalpy correction (H_CORR_) is -7.713±0.317 (n = 44). Hence, X-H BDG was ~5 kcal/mol lower than homolytic bond dissociation enthalpy (BDE). Here, we calculated X-H BDG instead of BDE for the unification of all energetic effects of dimerization reactions. For 2AP, L-3HOK and 3HAA, O-H BDE calculated at level II is ~71.0 kcal/mol (S3 Table), whereas the experimental 2AP O-H BDE is 81.3 kcal/mol [73]. The calculated O-H BDE of hydroxykynurenines may differ from the experimental absolute values, but their relative order is satisfactory reproduced by computational approach [32].

BSSE correction [74] was performed at level II for several calculations, including reactions with H-atom abstraction, as well as D1 formation from two monomers (A, B) separated by N-C bond (S3 Table). The negative BSSE correction decreases X-H BDG making it less positive and increases dimerization energy making it less negative. For D1 formation, BSSE correction is -5.373±2.904 kcal/mol (n = 4), being higher for the negatively charged D1-3HAAi form than for the neutral D1-3HAA form. For H-atom abstraction, BSSE correction values and data spread are significantly lower (-1.543±0.299 kcal/mol, n = 6). Thus, we did not consider BSSE while comparing X-H BDG values.

### Modeling Drosophila phenoxazinone synthase in complex with R-2AP

Homology modeling of *D*. *melanogaster* PHS in complex with the rigid heme group was performed with the help of MODELLER 9.14 software [75] using goat lactoperoxidase as a template (PDB ID: 2ojv, residues 13–596; 35% sequence identity). After the loops modeling, the model structure was dynamically optimized. The quality of the 10 model structures was estimated using SaliLab Model Evaluation Server [76–78]. The structure with minimal predicted RMSD (5.352 Å) between Cα atom coordinates in the native structure and the model, which also had the most negative value of MODELLER energy, was selected for docking. O_2_ position relative to heme was set the same as in human oxy-hemoglobin (PDB ID: 6bb5 A), except Fe-O distance was 1.95 Å instead of 1.85 Å. The partial charges for heme group were taken from [79]. For O_2_, the partial charges on atoms proximal and distal to Fe were -0.07 and 0.07, respectively. All the substrates were in the appropriate ionic form: 3HAA and its derivatives–the ionized COO^-^ group (3HAAi), L-3HOK and its derivatives–the ionized αCOO^-^ and αNH_3_^+^ groups (L-3HOKzi, zwitterion form). The optimal conformations were found using MMFF94 force field. The automatic docking of the flexible R-2AP substrates to the rigid PHS catalytic site was performed using Autodock 4.2 software [80] with the help of Lamarckian genetic algorithm [81]. PHS model and docked substrates are presented in S2 Dataset.

The illustrations were prepared using ChemSketch [82] and VMD [83].

### Total antioxidant capacity and lipid peroxidation measurement in Drosophila

#### *D*. *melanogaster s*trains

1. *Canton-S (CS);* Bloomington Drosophila Stock Center (BDSC), Indiana University, USA; #1. The wild-type strain.
2. *cardinal (cd[1])*; BDSC; #3052. Carries mutation in phenoxazinone synthase gene (FlyBase data); L-3НОК overproduction. The strain has been out-crossed to *CS* for 9 generations.

All strains were purchased from Biocollection of I.P. Pavlov Institute of Physiology of the Russian Academy of Sciences, Koltushi, St. Petersburg, Russia. The strains were maintained on the standard yeast–raisin medium at 22±0.5°C or 25±0.5°C and 12:12 daily illumination cycle.

#### Drosophila heads homogenization

Total antioxidant capacity (TAC) and lipid peroxidation (LP) were estimated in Drosophila heads at the 5^th^, 13^th^, 21^th^ and 29^th^ days of imaginal stage (n = 5 for different groups, 40–50 Drosophila heads in each sample). Drosophila heads were homogenized on ice using plastic pestle homogenizer in 600 ul solution containing 0.3 M sucrose (for the total antioxidant capacity assay but not for lipid peroxidation assay), 1mM EDTA, 0.2 M Tris-HCl, pH 7.4, and centrifuged for 10 minutes at 1000g and 4°C. The supernatant was recentrifuged for 20 minutes at 2000g and 4°C, similar to that in [84], or at 10000g for better purification of samples from insoluble debris which may affect the measurement. The obtained supernatant was frozen and stored at -70°C for further analysis of TAC and LP.

#### Total protein level assay

Protein content in the samples was measured before every experiment by a standard Eppendorf protocol using Biophotometer plus Eppendorf reader (Eppendorf, Germany) at the Research Centre for Environmental Safety of St. Petersburg State University. 5 μl of each sample was diluted to the final volume of 1 ml with distilled water, and the optical density was measured according to the standard Eppendorf protocol.

#### Measurement of the total antioxidant capacity

TAC was measured in Drosophila heads using oxygen radical absorbance capacity fluorimetric method. For this, 25 μl of sample was incubated with 7.5 nM fluorescein (Sigma Ald., USA) solution (10 mM potassium phosphate buffer, pH 7.4) for 30 minutes at 37°C. Then AAPH (Sigma Ald., USA) was added up to a final concentration of 30 mM [85]. Fluorescence was estimated by spectrofluorimetric microplate reader FLUOstar Omega (BMG Labtech, Germany) at the Research Centre for Environmental Safety of St. Petersburg State University (Ex. 485 nm, Em. 520 nm). To determine the range of hit of the studied samples in the linear range, a calibration curve was prepared using the standard antioxidant Trolox. The data were presented as a light sum per μg of total protein. For some pairs of experiments (with different fly keeping temperatures or centrifugation strengths), all values were normalized to the average for a given experiment, including both *CS* and *cd*, and merged into a total sample.

#### Measurement of the thiobarbituric acid reactive substances

Intensity of the LP in Drosophila heads was measured using thiobarbituric acid reactive substances (TBARS) colorimetric assay. For this, 200 μl of sample was incubated with 200 μl of 1% solution of orthophosphoric acid and 400 μl of 0.5% solution of thiobarbituric acid for 20 minutes at 100°C. The optical densities of the samples were measured by spectrophotometric microplate reader SPECTROstar Omega at wavelength 532 nm. The contribution of unspecific extinction by measuring the optical density was estimated at 600 nm [86]. The data were presented as TBARS fmol per μg of total protein.

### Statistical analysis

Pearson correlation and linear regression were calculated using Social Science Statistics online resource [87]. Statistical significance was estimated using two-sided randomization test at p <0.05 [88].

## Supporting information

S1 Table The average free energy differences for H-dissociation free energy (ΔG), IP, EA, and electronegativity (χ) (kcal/mol). X–C, O or N atom. Underlined: statistically significant differences (two-sided randomization test; p<0.05, n–see in Table).
(PDF)

S2 Table Pearson correlation between the energy values calculated for R-2AP and its derivatives. The values are given for the coefficient of determination R^2^. The name of 2AP, L-3HOK, 3HAA or 3HAAi stands for itself and all its dimeric forms. Underlined–non-significant correlation, *italics*–the moderate correlation.
(PDF)

S3 Table Free energies of dimerization, IP, EA, reaction rates and BSSE corrections calculated at the level II (B3LYP/6-31G(d,p). (XLS)

S1 Fig Homolytic X-H bond dissociation free energy (BDG) of R-2AP dimerization products. N–the number of reactions (according to Tables 1 and 2).
(TIFF)

S2 Fig Ionization potentials (IP) of R-2AP dimerization products. (TIFF)

S3 Fig Electron affinities (EA) of R-2AP dimerization products. (TIFF)

S1 Dataset The optimized structures of R-2AP dimers and complexes with molecular oxygen. Atom names, nucleus charges and coordinates (x, y, z; in Å) are given.
(TXT)

S2 Dataset Phenoxazinone structure and docked R-2AP compounds (pdb files). (ZIP)
