# Supplementary material for: Enzymatic and non-enzymatic pathways of kynurenines' dimerization: the molecular factors for oxidative stress development
Source: PLoS Comput Biol. 2018 Dec 10;14(12):e1006672. doi: 10.1371/journal.pcbi.1006672 (PMC6301705; doi:10.1371/journal.pcbi.1006672)
Supplement: S1 Table — X–C, O or N atom. Underlined: statistically significant differences (two-sided randomization test; p<0.05, n–see in Table). (PDF) [file pcbi.1006672.s001.pdf]

Compounds	Gas	Heptane	Water	n
		ΔG X-H		
3HAAi-D – 3HAA-D	-4.515±5.703	-2.765±5.618	0.960±3.814	11
2AP-D – L-3HOK-D	1.480±3.254	1.198±3.009	0.593±2.653	11
2AP-D – 3HAA-D	-2.849±2.494	-2.809±2.026	-2.303±1.328	11
		ΔG O-H and N-H		
3HAAi-D – 3HAA-D	-7.775±4.892	-5.080±4.930	-2.006±3.979	8
2AP-D – L-3HOK-D	2.560±4.251	2.315±3.754	1.774±3.278	8
2AP-D – 3HAA-D	-2.856±3.549	-2.905±2.766	-2.380±1.260	8
		ΔIP		
3HAAi-D – 3HAA-D	-135.900±15.423	-75.465±9.103	-7.662±4.075	15
2AP-D – L-3HOK-D	0.425±6.228	-2.762±4.512	-5.758±3.720	15
2AP-D – 3HAA-D	-4.372±3.678	-5.998±1.579	-6.476±1.696	15
		ΔEA		
3HAAi-D – 3HAA-D	-128.750±12.578	-71.785±6.227	-13.479±5.116	15
2AP-D – L-3HOK-D	-18.662±5.174	-14.879±6.379	-8.574±7.746	15
2AP-D – 3HAA-D	-13.892±4.569	-12.264±5.950	-10.094±6.036	15
		Δχ		
3HAAi-D – 3HAA-D	-132.325±13.450	-73.625±7.030	-10.570±2.172	15
2AP-D – L-3HOK-D	-9.253±4.323	-8.820±4.203	-7.166±4.098	15
2AP-D – 3HAA-D	-9.132±2.846	-9.131±2.849	-8.285±2.771	15
