# Supplementary material for: Enzymatic and non-enzymatic pathways of kynurenines' dimerization: the molecular factors for oxidative stress development
Source: PLoS Comput Biol. 2018 Dec 10;14(12):e1006672. doi: 10.1371/journal.pcbi.1006672 (PMC6301705; doi:10.1371/journal.pcbi.1006672)
Supplement: S2 Table — The values are given for the coefficient of determination R2. The name of 2AP, L-3HOK, 3HAA or 3HAAi stands for itself and all its dimeric forms. Underlined–non-significant correlation, italics–the moderate correlation. (PDF) [file pcbi.1006672.s002.pdf]

I Free energy correlation (n=21, some related values are omitted)

		Gas				Heptane				Water			
		2AP	L-3HOK	3HAA	3HAAi	2AP	L-3HOK	3HAA	3HAAi	2AP	L-3HOK	3HAA	3HAAi
Gas	2AP		0.970	0.973	<u>0.016</u>	0.930				0.804			
	L-3HOK			0.959	<u>0.002</u>		0.941				0.829		
	3HAA				<u>0.037</u>			0.943				0.852	
	3HAAi								0.861				<u>0.189</u>
Heptane	2AP						0.965	0.980	*0.447*	0.963			
	L-3HOK							0.958	*<u>0.338</u>*		0.966		
	3HAA								*0.496*			0.975	
	3HAAi												*0.540*
Water	2AP										0.971	0.990	0.939
	L-3HOK											0.971	0.938
	3HAA												0.947
	3HAAi												

II. Ionization potential correlation (n = 15)

		Gas				Heptane				Water			
		2AP	L-3HOK	3HAA	3HAAi	2AP	L-3HOK	3HAA	3HAAi	2AP	L-3HOK	3HAA	3HAAi
Gas	2AP		0.847	0.934	*0.543*	0.979				0.957			
	L-3HOK			0.952	*0.363*		0.926				0.899		
	3HAA				*0.481*			0.990				0.957	
	3HAAi								0.830				*0.527*
Heptane	2AP						0.754	0.970	0.617	0.987			
	L-3HOK							0.838	*0.353*		0.887		
	3HAA								*0.551*			0.983	
	3HAAi												0.670
Water	2AP										0.766	0.954	0.790
	L-3HOK											0.906	0.603
	3HAA												0.751
	3HAAi												

III Electron affinity correlation (n = 15)

		Gas				Heptane				Water			
		2AP	L-3HOK	3HAA	3HAAi	2AP	L-3HOK	3HAA	3HAAi	2AP	L-3HOK	3HAA	3HAAi
Gas	2AP		0.946	0.939	<u>0.122</u>	0.989				0.971			
	L-3HOK			0.948	<u>0.057</u>		0.982				0.801		
	3HAA				<u>0.095</u>			0.977				0.952	
	3HAAi								*0.546*				<u>0.148</u>
Heptane	2AP						0.966	0.941	0.686	0.994			
	L-3HOK							0.947	0.637		0.840		
	3HAA								0.678			0.973	
	3HAAi												0.724
Water	2AP										0.867	0.960	0.956
	L-3HOK											0.892	0.814
	3HAA												0.956
	3HAAi												

IV. Electronegativity correlation (n = 15)

		Gas				Heptane				Water			
		2AP	L-3HOK	3HAA	3HAAi	2AP	L-3HOK	3HAA	3HAAi	2AP	L-3HOK	3HAA	3HAAi
Gas	2AP		0.921	0.960	*0.294*	0.980				0.964			
	L-3HOK			0.955	0.126		0.966				0.937		
	3HAA				0.256			0.994				0.993	
	3HAAi								0.812				0.242
Heptane	2AP						0.910	0.965	*0.556*	0.996			
	L-3HOK							0.929	*0.445*		0.926		
	3HAA								0.580			0.991	
	3HAAi												*0.551*
Water	2AP										0.958	0.962	0.968
	L-3HOK											0.958	0.908
	3HAA												0.956
	3HAAi												

V. Energy values calculated at level II and level III (Gas)

	Level II (x)	Level III Gas (y)			
		2AP	L-3HOK	3HAA	3HAAi
ΔG<br><br>n=23	2AP	0.998			
	L-3HOK		0.997		
	3HAA			0.998	
	3HAAi				0.993
IP<br><br>n=15<br>(16 for L-3HOK)	2AP	1.000			
	L-3HOK		0.999		
	3HAA			1.000	
	3HAAi				0.966
EA<br><br>n=15<br>(16 for L-3HOK)	2AP	0.996			
	L-3HOK		0.999		
	3HAA			0.993	
	3HAAi				0.767

For ΔG: all stages except N26; N7 and N8 stages are united, as well as N13 and N14.

VI. Log k(T) values calculated at level II and level III (Gas, Heptane, Water) (n = 20).

	III (Gas)	III (Heptane)	IV (Water)
II	0.973	0.963	0.956
III (Gas)		0.993	0.974
III (Heptane)			0.979
