# Supplementary figures and images for: Enzymatic and non-enzymatic pathways of kynurenines' dimerization: the molecular factors for oxidative stress development
Source: PLoS Comput Biol. 2018 Dec 10;14(12):e1006672. doi: 10.1371/journal.pcbi.1006672 (PMC6301705; doi:10.1371/journal.pcbi.1006672)

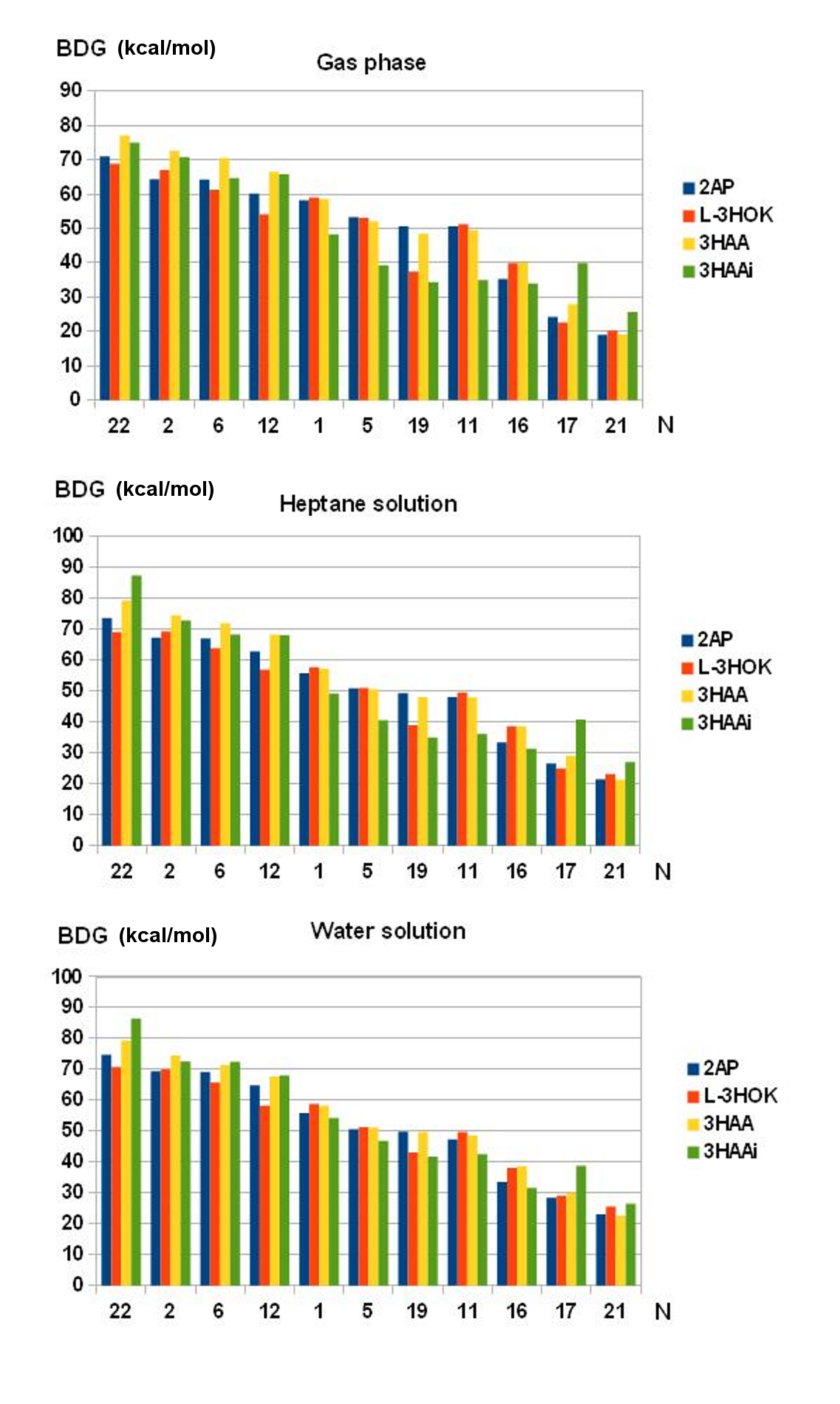

Supplement: S1 Fig — N–the number of reactions (according to Tables 1 and 2). (TIFF) [file pcbi.1006672.s004.tiff]

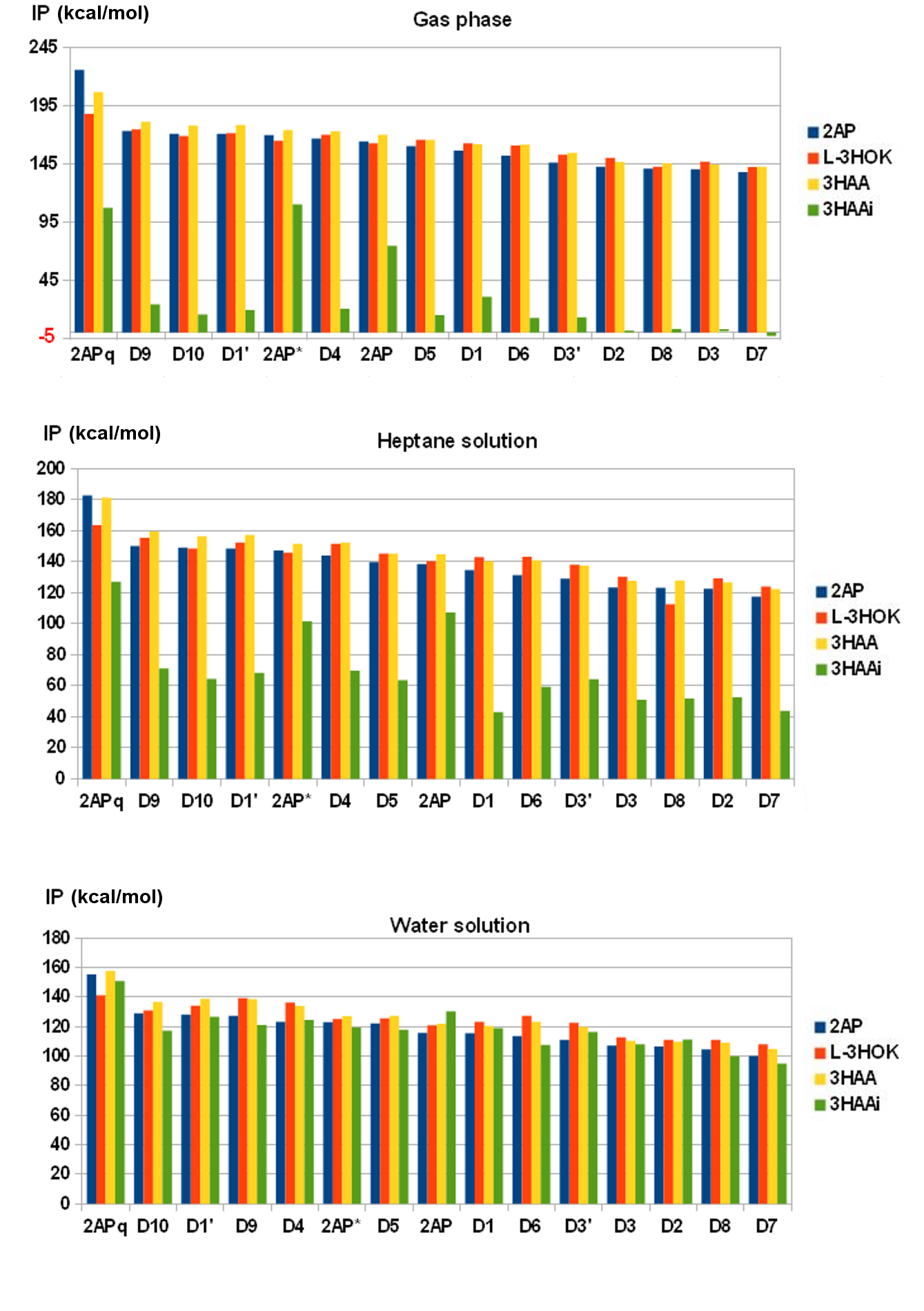

Supplement: S2 Fig — (TIFF) [file pcbi.1006672.s005.tiff]

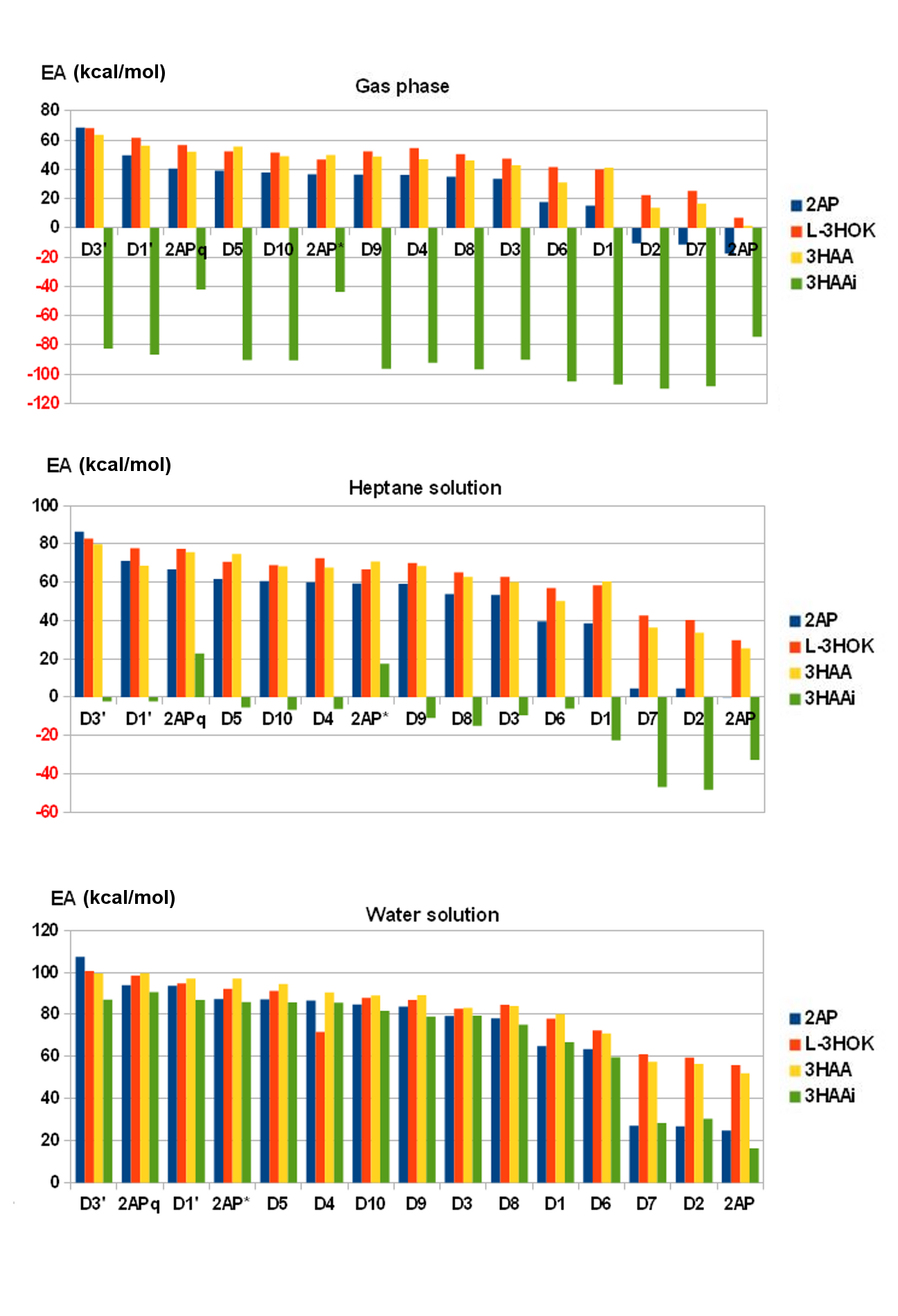

Supplement: S3 Fig — (TIFF) [file pcbi.1006672.s006.tiff]
